# The role of ferroptosis-related genes in airway epithelial cells of asthmatic patients based on bioinformatics

**DOI:** 10.1097/MD.0000000000033119

**Published:** 2023-03-03

**Authors:** Ye Zheng, Jingyao Fan, Xiaofeng Jiang

**Affiliations:** a Department of Clinical Laboratory, The Fourth Affiliated Hospital of Harbin Medical University, Harbin, China; b Department of Clinical Laboratory, The Second Affiliated Hospital of Harbin Medical University, Harbin, China.

**Keywords:** airway epithelial cells, asthma, biomarker, drug, ferroptosis, molecular mechanisms

## Abstract

It has been reported that airway epithelial cells and ferroptosis have certain effect on asthma. However, the action mechanism of ferroptosis-related genes in airway epithelial cells of asthmatic patients is still unclear. Firstly, the study downloaded the GSE43696 training set, GSE63142 validation set and GSE164119 (miRNA) dataset from the gene expression omnibus database. 342 ferroptosis-related genes were downloaded from the ferroptosis database. Moreover, differentially expressed genes (DEGs) between asthma and control samples in the GSE43696 dataset were screened by differential analysis. Consensus clustering analysis was performed on asthma patients to classify clusters, and differential analysis was performed on clusters to obtain inter-cluster DEGs. Asthma-related module was screened by weighted gene co-expression network analysis. Then, DEGs between asthma and control samples, inter-cluster DEGs and asthma-related module were subjected to venn analysis for obtaining candidate genes. The last absolute shrinkage and selection operator and support vector machines were respectively applied to the candidate genes to screen for feature genes, and functional enrichment analysis was performed. Finally, a competition endogenetic RNA network was constructed and drug sensitivity analysis was conducted. There were 438 DEGs (183 up-regulated and 255 down-regulated) between asthma and control samples. 359 inter-cluster DEGs (158 up-regulated and 201 down-regulated) were obtained by screening. Then, the black module was significantly and strongly correlated with asthma. The venn analysis yielded 88 candidate genes. 9 feature genes (NAV3, ITGA10, SYT4, NOX1, SNTG2, RNF182, UPK1B, POSTN, SHISA2) were screened and they were involved in proteasome, dopaminergic synapse etc. Besides, 4 mRNAs, 5 miRNAs, and 2 lncRNAs collectively formed competition endogenetic RNA regulatory network, which included RNF182-hsa-miR-455-3p-LINC00319 and so on. The predicted therapeutic drug network map contained NAV3-bisphenol A and other relationship pairs. The study investigated the potential molecular mechanisms of NAV3, ITGA10, SYT4, NOX1, SNTG2, RNF182, UPK1B, POSTN, SHISA2 in airway epithelial cells of asthmatic patients through bioinformatics analysis, providing a reference for the research of asthma and ferroptosis.

## 1. Introduction

Bronchial asthma is a common chronic inflammatory disease worldwide, which affects more than 300 million people and brings a heavy burden to the public healthcare system.^[1,2]^ It is induced by allergens, and is accompanied by cough, shortness of breath, and wheezing. Repeated inflammatory stimulation of bronchial asthma can lead to airway hyperresponsiveness, airway remodeling, and mucus secretion.^[3]^

Airway epithelial cells are the primary barrier to allergens, and involved in the development of asthma. Firstly, airway epithelial cells can clear allergens through mucociliary clearance mechanisms.^[3]^ Mucin dysregulation has been observed in asthmatic patients. Secondly, airway epithelial cells can recognize pathogen/danger-associated molecular patterns through a variety of pattern recognition receptors, and then they can secrete alarmin cytokines (IL25, IL33, and TSLP) and chemokines (CCL5, CCL7, and CCL22), causing immune cell infiltration via chemotactic effects and resulting in local immune response.^[4–8]^ Thirdly, they also act as antigen-presenting cells, present antigen peptides, and induce the differentiation of naive T cells into CD4^+^ T cells, which plays a pivotal role in type II immune response.^[9,10]^ To date, focusing on adaptive immune resistant components in asthmatic patients has not fully cured asthma. Thus, we hypothesize that airway epithelial cell defects may also contribute to asthma.

Ferroptosis, a non-apoptosis regulated cell death characterized by iron-dependent lipid peroxidation and reactive oxygen species (ROS) accumulation, has significantly different morphological characteristics, biochemical indicators, and genetics than other known cell death patterns.^[11–13]^ In ferroptosis, the cell membrane is intact, and the nuclear morphology is not altered. However, the mitochondrial function is significantly impaired, the raw materials for intracellular synthesis of glutathione (GSH) are reduced, the glutathione peroxidase 4 is inactivated, and ROS in the cytoplasm is increased.^[14]^ In the lung tissue of mice with house dust mite induced asthma, the glutathione peroxidase 4 and solute carrier family 7 member 11 (SLC7A11) which is an important negative regulator of ferroptosis were significantly decreased, whereas ROS levels were significantly increased, compared to normal controls. Additionally, IL4, IL13, and IL33 produced by airway epithelial cells were increased in the bronchoalveolar lavage fluid of asthmatic mice.^[15]^ Treatment with ferroptosis inhibitors ferrostatin-1 (Fer-1) could alleviate airway inflammation and reduce airway epithelial cell death, indicating that airway epithelial cell ferroptosis is closely related to asthma.^[16]^ However, in human airway epithelial cells, the genes significantly related to ferroptosis of airway epithelial cells are unknown, and whether ferroptosis genes can be used as biomarkers to discriminate normal and asthmatic patients has not yet been reported.

Therefore, this study identified 9 feature genes significantly related to ferroptosis and asthma in human airway epithelial cells, and their ability to diagnose asthma, regulatory networks, biological functions, and therapeutic drug network were analyzed. Our findings may provide new direction for the pathogenesis and treatment of asthma.

## 2. Material and methods

### 2.1. Data source

This analysis downloaded the GSE43696 training set (Asthma: Control = 88: 20), GSE63142 validation set (Asthma: Control = 128: 27), and GSE164119 (miRNA) (Asthma: Control = 9: 7) datasets from gene expression omnibus database (https://www.ncbi.nlm.nih.gov/gds). Among them, GSE164119 (miRNA) and GSE43696 datasets were respectively used for the identification of differentially expressed miRNA (DE-miRNA) and differentially expressed lncRNA (DE-lncRNA). 342 ferroptosis-related genes (FRGs) were downloaded from ferroptosis database (FerrDb; http://www.zhounan.org/ferrdb/current/).

### 2.2. Acquisition of differentially expressed FRGs (DE-FRGs)

Based on GSE43696 dataset, differentially expressed genes (DEGs) between asthma samples and control samples were screened by limma package setting the condition of |log_2_FC| > 0.5 and *P* < .05.^[17]^ The volcano map was plotted based on the obtained results. DEGs of Top100 were displayed by drawing a heat map with the pheatmap package.^[18]^ Then, venn analysis of DEGs and FRGs was performed to obtain DE-FRGs.

### 2.3. Enrichment analysis of DE-FRGs

The gene ontology (GO) system included biological process (BP), molecular functions (MF), and cellular components (CC). Kyoto encyclopedia of genes and genomes (KEGG) database comprehensively integrated genomic, proteomic, chemical components, and other systematic functional information. This study used clusterprofiler package to perform GO and KEGG enrichment analysis on DE-FRGs (*P* < .05). The enrichment results were presented using the ggplot2 package.^[19,20]^

### 2.4. Construction of the protein-protein interaction (PPI) network

In order to explore whether there was a reciprocal relationship among DE-FRGs, we used STRING (https://string-db.org) website to set confidence = 0.4 to remove discrete proteins and obtain a PPI network. Moreover, based on GSE43696 dataset, the receiver operating characteristic (ROC) curve was drawn through pROC package to explore diagnostic power of key genes.^[21]^ Then, the assessment results were validated by GSE63142 dataset.

### 2.5. Consensus clustering analysis

The study used consensusclusterplus package to perform consensus clustering analysis on 88 asthma patients according to DE-FRGs.^[22]^ The number of clusters k was chosen to classify asthma patients, and principal component analysis was performed on the clusters to clarify their distribution. The differential analysis was performed on clusters by limma package setting |log_2_fold change| > 0.5 and *P* < .05 to screen out inter-cluster DEGs.^[17]^ In addition, the box plot was drawn to show the expression trends of DE-FRGs in different clusters.

### 2.6. Functional enrichment analysis of inter-cluster DEGs

Firstly, we respectively used c2.cp.kegg.v7.2.symbols.gmt and c5.go.bp.v7.5.1.symbols.gmt as the reference gene sets. Based on the gene expression profile files, the GO and KEGG pathways was scored in each sample by GSVA package.^[23]^ Then, GO and KEGG entries with inter-cluster differences were obtained by limma package setting the |log_2_(fold change)| > 0.1 and *P* < .05. The expression heat map was plotted by pheatmap package to display the results. In addition, GO and KEGG pathways on inter-cluster DEGs were performed using the clusterprofiler package. The enrichment results were visualized by ggplot2 package.

### 2.7. Weighted gene co-expression network analysis (WGCNA)

In this study, all gene expression matrices of 108 samples (Asthma: Control = 88: 20) in the GSE43696 dataset were treated as input data by WGCNA package.^[24]^ Asthma and control were used as trait to construct a co-expression network. Next, in order to ensure the accuracy of the analysis, the samples were clustered for excluding the outliers. Sample clustering and clinical trait heat map were constructed to visualize the results. A soft threshold was determined for the data to ensure that the interactions among genes were maximally consistent with the scale-free distribution. The modules were segmented by dynamic tree cutting algorithm setting minModuleSize = 300. Finally, we performed correlation analysis between gene modules and asthma, and selected the module which was most related to the disease as the asthma-related module.

### 2.8. Delineation of gene network clusters

Gene pairs in modules which was significantly strongly correlated with asthma were found using the STRING (https://string-db.org) website, and they were imported into cytoscape software. Gene network clusters were classified by molecular complex detection (MCODE) with the Degree Cutoff = 2, Node score cutoff = 2, k-score = 2 and Max.Depth = 100. The gene clusters of MCODE score top5 were selected for display.

### 2.9. Screening out feature genes by machine learning method

First, we performed venn analysis on DEGs between asthma and control samples, inter-cluster DEGs and asthma-related module to obtain candidate genes. Then, least absolute shrinkage and selection operator (LASSO) regression analysis was performed on the candidate genes by glmnet package to obtain gene coefficient map and cross-validation error map.^[25]^ The support vector machines (SVM)-recursive feature elimination (RFE) algorithm was acted on the candidate genes to narrowed down the range of feature genes. Finally, the intersection of the genes screened by LASSO and SVM algorithms was taken to obtain the feature genes. The GSE43696 dataset contained 50 mild to moderate asthma samples, 38 severe asthma samples and 20 healthy samples. We divided the GSE43696 dataset into 2 small datasets of 50 mild to moderate asthma samples versus 20 healthy samples and 38 severe asthma samples versus 20 healthy samples. In order to test whether the feature genes had ability to identify different levels of asthma, the ROC analysis was made for two small datasets, and the GSE63142 dataset was used to verify the results.

### 2.10. Construction of a nomogram and gene set enrichment analysis (GSEA) of feature genes

Based on the GSE43696 dataset, we used rms package to construct the diagnostic nomogram of feature genes and clinical factors.^[26]^ Scores for age and gender clinical factors were calculated, and the scores for each factor were summed to obtain total points, then, the survival rate of patients was predicted based on the total points. Calibration curve was plotted to verify the validity of nomogram. Besides, the feature genes were correlated with all genes of the asthma samples in turn and ranked. The screening conditions for GSEA were SIZE > 20 and NOM.*P* < .05.

### 2.11. Screening for DE-miRNA and DE-lncRNA

Differential analysis was performed on 7 control samples and 9 asthma samples in the GSE164119 dataset using the edgeR package.^[27]^ Differential analysis of 20 control samples and 88 asthma samples in the GSE43696 dataset was carried out by limma package. The screening criteria for DE-miRNA and DE-lncRNA were |log_2_fold change| > 0.5 and *P* < .05. Volcano plot and heat map were drawn to visualize the results.

### 2.12. Construction of a competing endogenous RNA (ceRNA) regulatory network

ceRNA is a role element that can compete for binding RNA. We used miRWalk website (http://mirwalk.umm.uni-heidelberg.de/) to predict the miRNAs of up-regulated feature genes with default parameters score = 0.95 and position = 3UTR. The miRNAs were intersected with the down-regulated miRNAs to obtain the miRNA1. lncRNA1 which interacted with miRNA1 was forecasted using the starBase database. The genes with regulatory relationships were extracted based on the miRNA1. Then, miRWalk was used to predict the miRNAs of down-regulated feature genes, and the miRNAs were intersected with the up-regulated miRNAs to obtain the miRNA 2. lncRNA2 that interacted with miRNA2 was predicted through starBase database. The shared lncRNAs were obtained by taking intersection of lncRNA 2 and down-regulated lncRNAs, then, the miRNAs and genes with regulatory relationships were extracted from the shared lncRNAs. Finally, mRNA-miRNA-lncRNA interaction pairs were obtained by integrating the prediction results of up- and down-regulated feature gene. The interaction relationships were visualized using the cytoscape software.

### 2.13. Construction of the mRNA-transcription factor (TF) regulatory network

In this study, we used NetworkAnalyst (https://www.networkanalyst.ca/) database to retrieve the TFs which regulated feature genes. A mRNA-TF regulatory network was constructed using cytoscape software on feature genes and TFs. To explore the correlation between the feature genes and TF, their correlation coefficients and *P* value were calculated by spearman. The results were displayed by drawing correlation heat map.

### 2.14. Prediction of potential therapeutic drugs

The feature genes were entered in CTD database (http://ctdbase.com/) to predict potential therapeutic agents for the treatment of asthma patients. The screening condition was Interaction Count ≥ 2.

## 3. Results

### 3.1. Identification and functional annotation analysis of DE-FRGs

**Figure 1.**
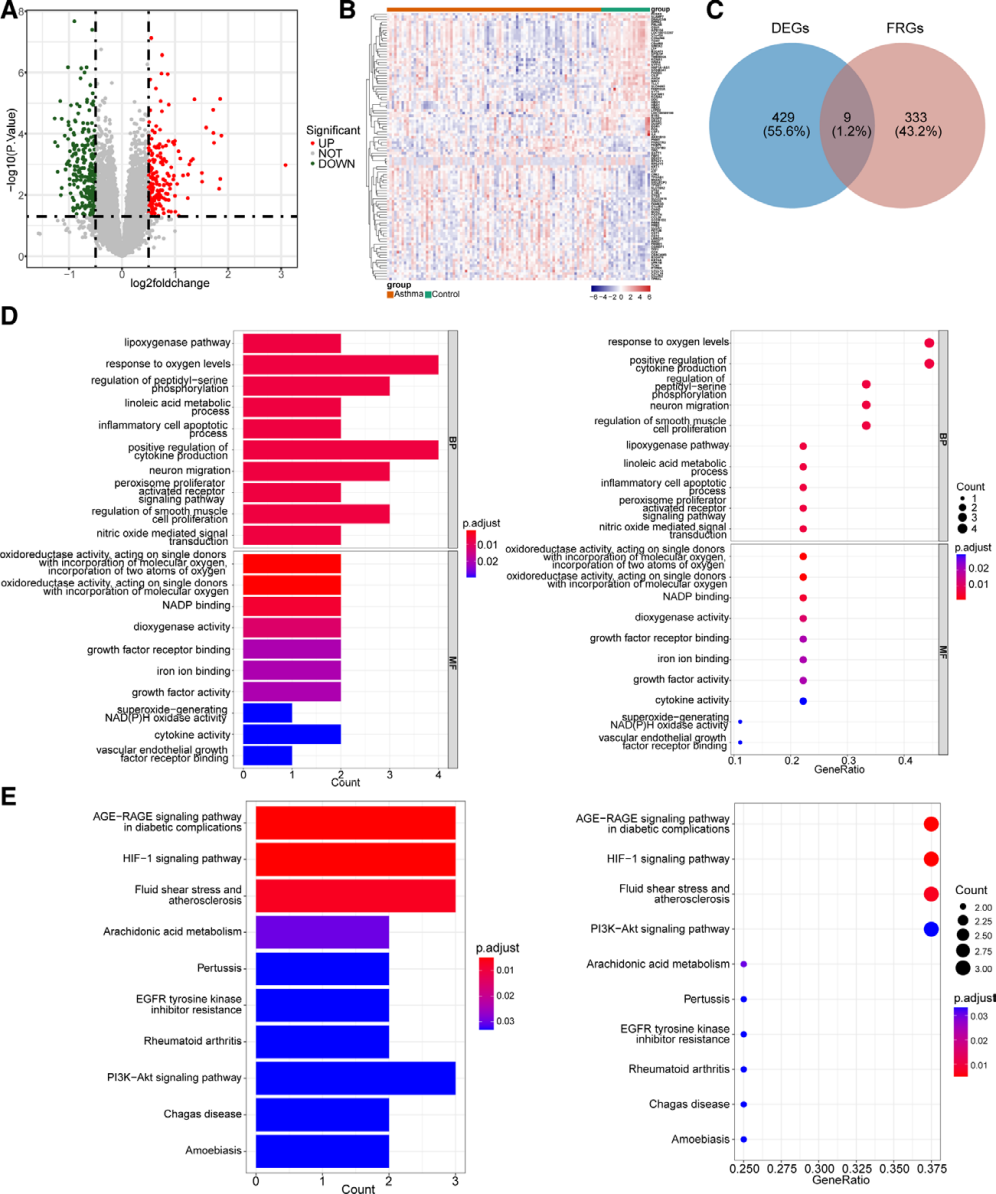
Identification and functional annotation analysis of DE-FRGs. (A) Volcano plot of differential genes of asthma and normal control subjects. (B) Heat map of up and down Top100 differential genes of asthma and normal control subjects. Each small square represents each gene, and its color represents the expression level of the gene. The dendrogram on the left represents the cluster analysis results of different genes from different samples. (C) Venn diagram of DEGs and ferroptosis-related genes (FRGs). (D) GO function annotation results (Top10). (E) KEGG pathway enrichment results (Top10). DE-FRGs = differentially expressed FRGs, DEGs = differentially expressed genes, GO = gene ontology, KEGG = Kyoto encyclopedia of genes and genomes.

There were 438 DEGs (183 up-regulated DEGs and 255 down-regulated DEGs) between asthma and control samples. The results were visualized in heat and volcano plots (Fig. 1 A and B). 9 DE-FRGs were differentially expressed between asthma and control samples (Fig. 1C). DE-FRGs were involved in 575 BP, 20 MF, and 13 KEGG signaling pathways, of which BP included the lipoxygenase pathway, response to oxygen levels, regulation of peptidyl-serine phosphorylation and others, KEGG pathways contained AGE-RAGE signaling pathway in diabetic complications, fluid shear stress and atherosclerosis, HIF-1 signaling pathway, etc. The bubble diagram and bar graph displayed a part of enriched GO and KEGG pathways (Fig. 1D and E).

### 3.2. Identification and evaluation of the key genes

**Figure 2.**
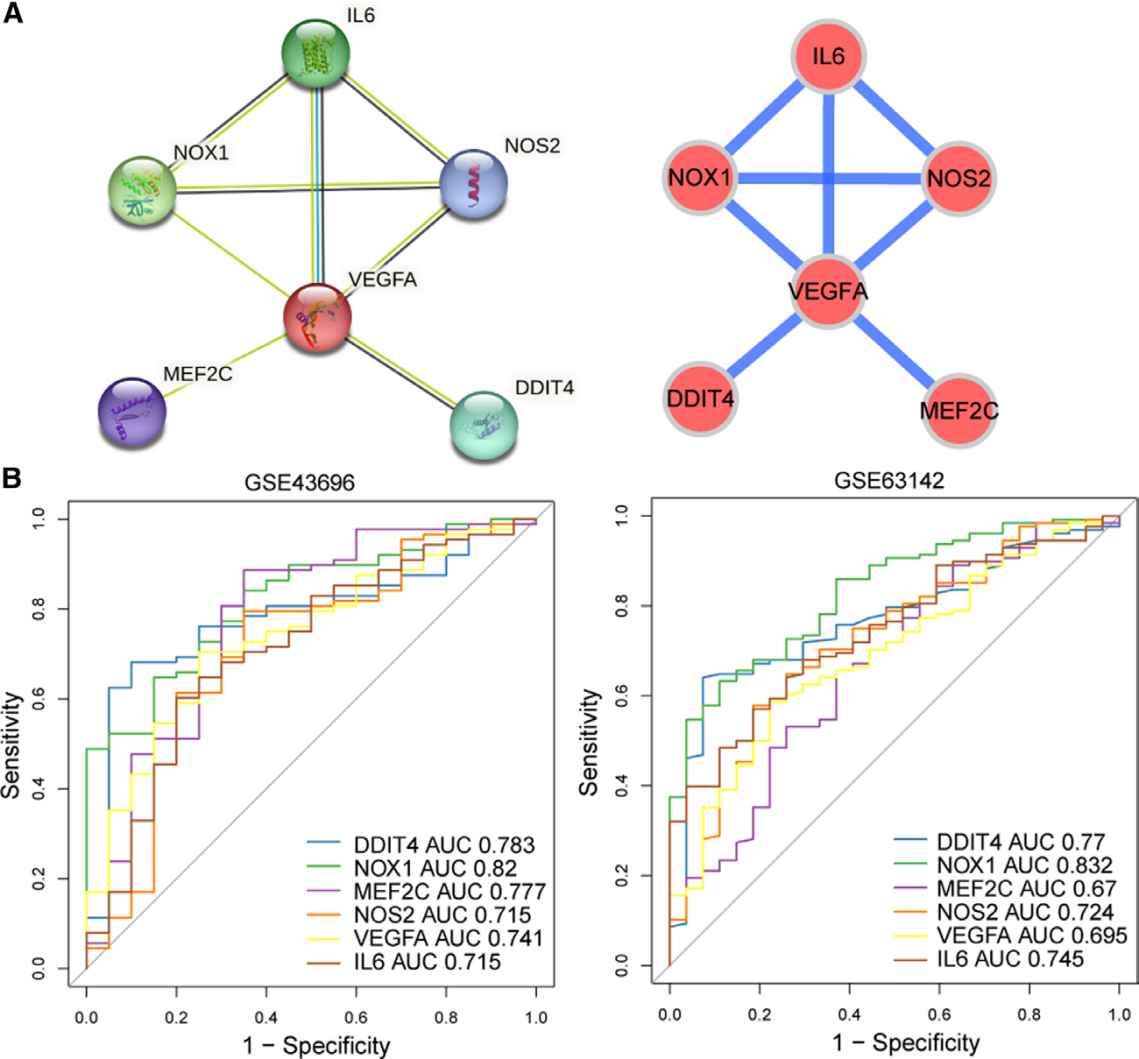
Identification and evaluation of the key genes. (A) The PPI network downloaded from the STRING database indicated the interactions among the 6 DE-FRGs. (B) ROC curves of 6 DE-FRGs in training set (GSE43696) and validation set (GSE63142). DE-FRGs = differentially expressed FRGs, PPI = protein-protein interaction, ROC = receiver operating characteristic.

A total of 6 nodes and 8 edges constituted the PPI network, which included IL6-NOX1, VEGFA-DDIT4, etc. (Fig. 2A). The 6 obtained genes namely IL6, NOX1, VEGFA, DDIT4, MEF2C, NOS2 were taken as key genes. From the ROC curve of the GSE43696 dataset, we could see that area under the curve (AUC) of DDIT4 = 0.783, NOX1 AUC = 0.82, MEF2C AUC = 0.777, NOS2 AUC = 0.715, VEGFA AUC = 0.741, and IL6 AUC = 0.715, their AUC values were all greater than 0.7, indicating that the key genes had a better ability to distinguish asthma and control. In the ROC curve of the GSE63142 validation set, the AUC of the key genes were all exceeded 0.6, similarly validating a good diagnostic ability of their (Fig. 2B).

### 3.3. Classification of asthma patient clusters and identification of inter-cluster DEGs

**Figure 3.**
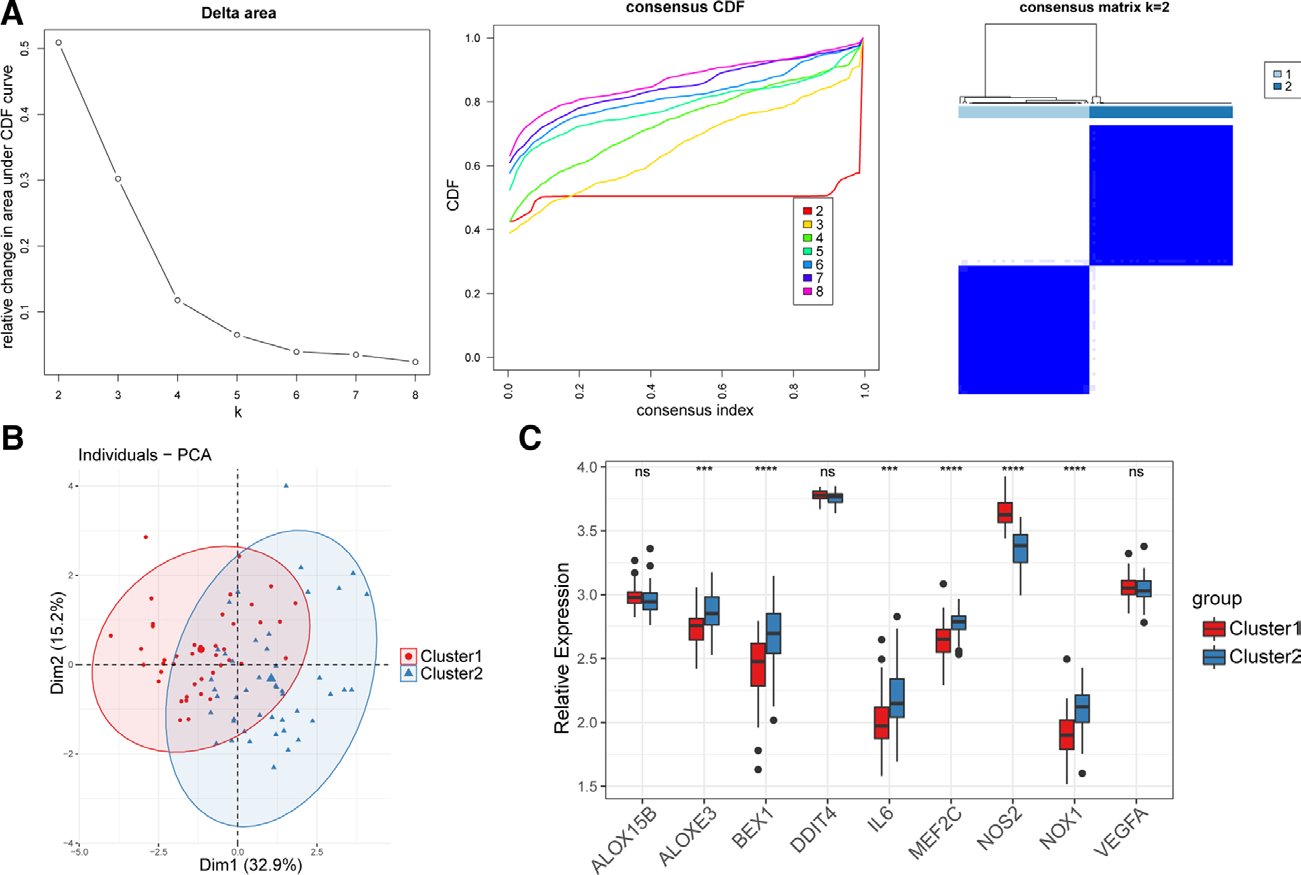
Classification of asthma patient clusters and identification of inter-cluster DEGs. (A) Consensus Cluster analysis identified two distinct subtypes of asthma subjects according to DE-FRGs within the training set. (B) Graph of PCA score plot for cluster 1 and cluster 2 of asthma subjects. (C) Expression of 9 DE-FRGs in cluster 1 and cluster 2. CDF= cumulative distribution function, DE-FRGs = differentially expressed FRGs, DEGs = differentially expressed genes, GSVA= gene set variation analysis, PCA = principal component analysis.

Asthma patients were divided into cluster 1 (N = 42) and cluster 2 (N = 46) according to 9 DE-FRGs by consensus clustering analysis (Fig. 3A). The results of principal component analysis showed that 2 clusters were clearly distributed in different regions (Fig. 3B). A total of 359 inter-cluster DEGs (158 were up-regulated and 201 were down-regulated) were obtained after the screening. It could be seen from the box plot that the expression of ALOXE3, BEX1, IL6, MEF2C, NOS2, and NOX1 were extremely significant different between cluster 1 and cluster 2 (Fig. 3C).

### 3.4. Functional annotation analysis of inter-cluster DEGs

**Figure 4.**
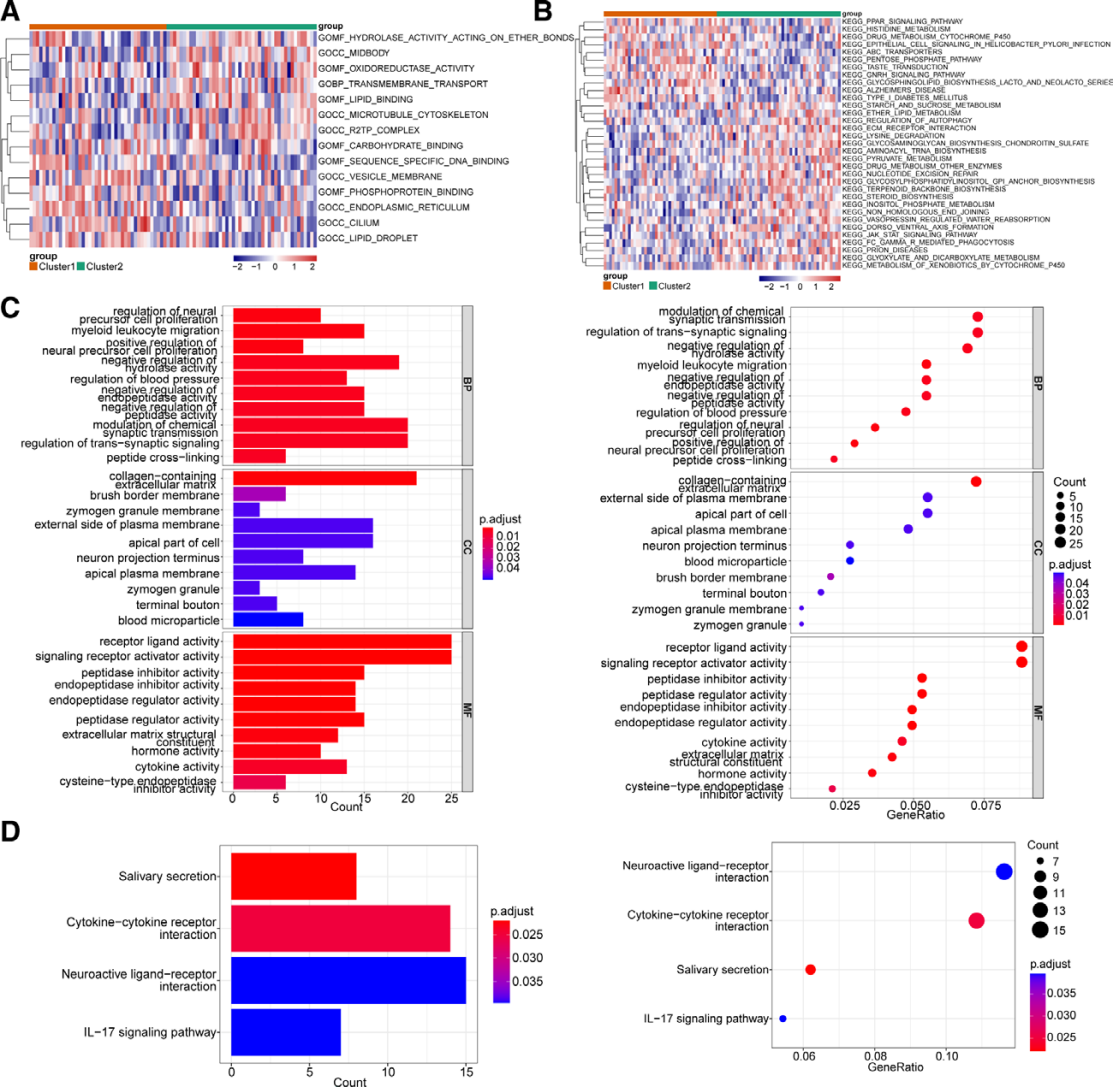
Functional annotation analysis of inter-cluster DEGs. (A) Heatmap of GSVA score for GO function annotation of cluster 1 and cluster 2. (B) Heatmap of GSVA score for KEGG enrichment of cluster 1 and cluster 2. (C) GO function annotation in differential genes of cluster 1 and cluster 2. (D) KEGG pathway enrichment results in differential genes of cluster 1 and cluster 2. DEGs = differentially expressed genes, GO = gene ontology, KEGG = Kyoto encyclopedia of genes and genomes.

There were 14 GO entries and 33 KEGG pathways between cluster 1 and cluster 2. GO entries included hydrolase activity acting on ether bonds, midbody, oxidoreductase activity, etc., and KEGG signaling pathways contained ppar signaling pathway, histidine metabolism, drug metabolism cytochrome P450 and others (Fig. 4A and B). In addition, a total of 63 BP, 10 CC, 12 MF, and 4 KEGG enrichment pathways were enriched by inter-cluster DEGs, in which the BP pathways covered regulation of neural precursor cell proliferation, myeloid leukocyte migration and others. KEGG signaling pathways consisted of salivary secretion, cytokine-cytokine receptor interaction, neuroactive ligand-receptor interaction and IL-17 signaling pathway. The bar and bubble diagrams showed the GO and KEGG enrichment pathways (Fig. 4C and D).

### 3.5. Screening of the gene module

**Figure 5.**
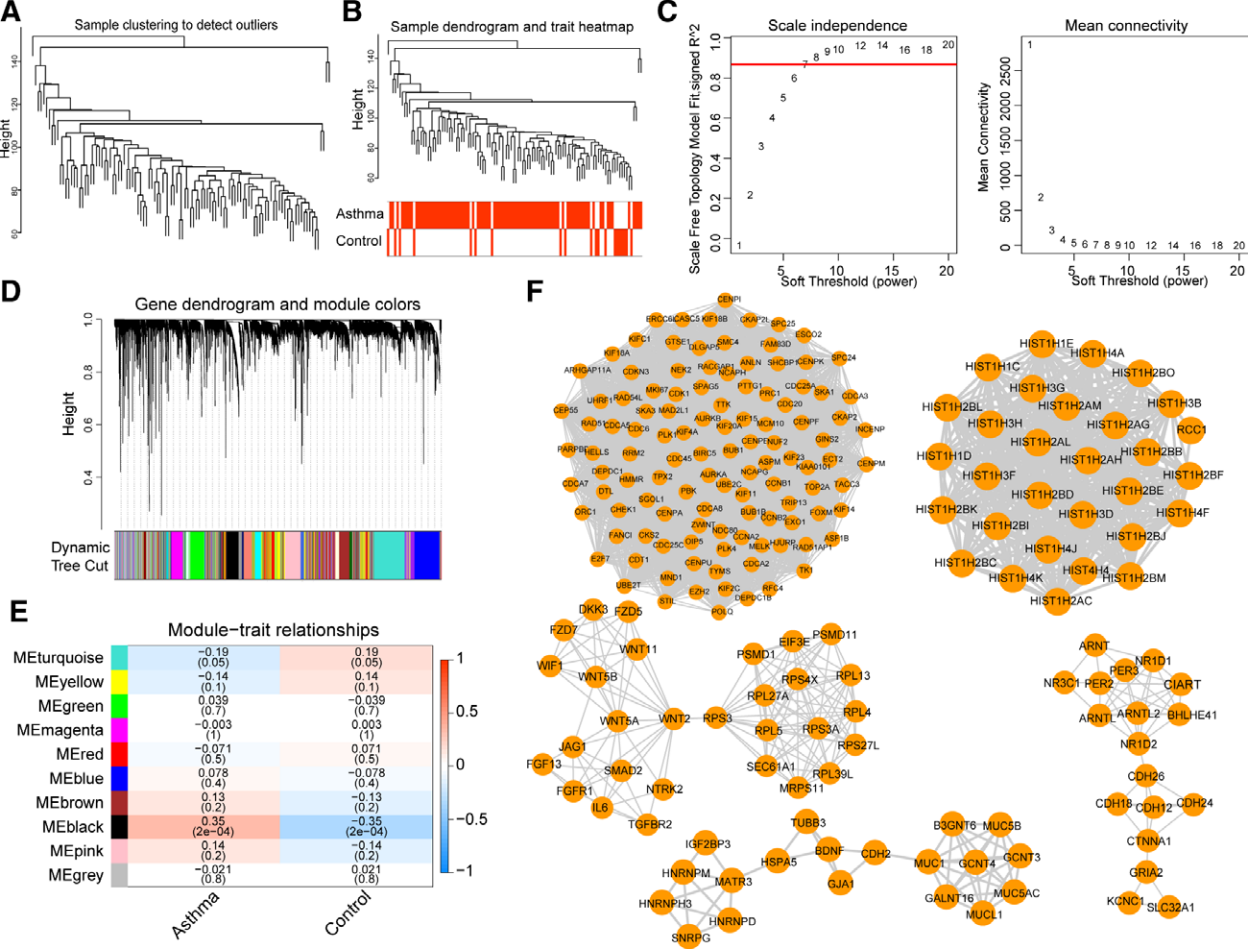
Screening of the gene module by WGCNA. (A) Clustering information of GSE43696 samples. (B) Clustering and phenotypic information of GSE43696 samples, the upper part of the figure is clustering and the lower part is group. (C) Determination of the soft threshold. (D) Merging of the similar modules analyzed by the dynamic cutting tree algorithm (minModuleSize = 300). (E) The heat map of correlation between different modules and clinical characters. The ordinate represents different modules and the abscissa represents different groups. Each block represents the correlation coefficient and significance *P* value of a module and group. (F) The five top score clusters of genes/proteins related to asthma based on the black module. WGCNA = Weighted gene co-expression network analysis.

From Figure 5A, it could be seen that the overall clustering of the dataset samples was good and no sample elimination was required. The sample clustering and clinical trait heat maps were presented for the results (Fig. 5B). The optimal soft threshold was determined to be 7 by WGCNA, and the *R*^2^ was around 0.87 at this time, indicating that the network approximated a scale-free network (Fig. 5C). A series of 10 gene modules were screened by constructing co-expression matrices (Fig. 5D). The heat map showed that the black module (1404 genes) was significantly and strongly correlated with asthma (Fig. 5E). The black module was divided into 42 gene network clusters, including SNRPG-HNRNPD, GJA1-CGH2 and other gene relationship pairs (Fig. 5F).

### 3.6. Identification and evaluation of feature genes

**Figure 6.**
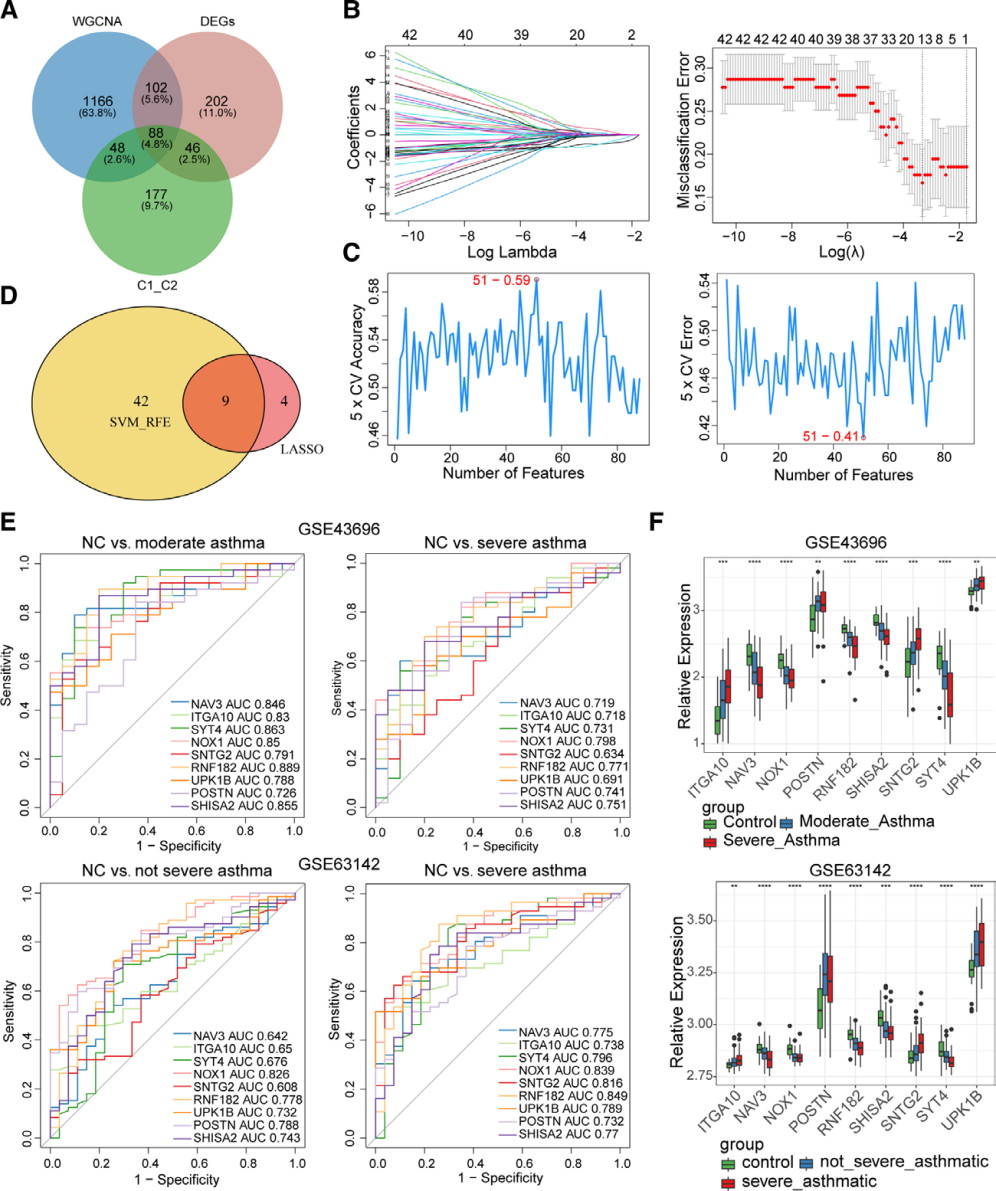
Identification feature genes by machine learning method and feature genes evaluation. (A) Venn plot exhibiting the reliable gene features about asthma and ferroptosis among WGCNA (MEblack), DEGs, and two clusters’ DEGs. (B) LASSO feature selection method to narrowed down the 88 gene features. The left picture is convergence graph of 88 gene features. Each curve represents the coefficient change trajectory of each independent variable. The right picture is adjusting the different parameters to achieve the minimum binomial deviation of the model based on the penalty parameter that was determined by 10-fold cross validation following the 1-SE criterion, the best-performing feature set was selected. (C) SVM-RFE algorithm to narrowed down the 88 gene features. The pictures show the average accuracy/error as a function of the number of selected gene features. (D) Venn diagram of SVM_RFE and LASSO. (E) ROC curves of 9 feature genes related ferroptosis to distinguish between normal control and asthmatic subjects in GSE43696 and GSE63142. (F) Expression of 9 feature genes in normal control and asthmatic subjects. DEGs = differentially expressed genes, LASSO = least absolute shrinkage and selection operator, NC = normal control, ROC = receiver operating characteristic, RFE = recursive feature elimination, SVM = support vector machines, WGCNA = Weighted gene co-expression network analysis.

A total of 88 candidate genes were obtained through venn analysis (Fig. 6A). 13 genes (NAV3, ITGA10, CSH1, SYT4, NOX1, SNTG2, LRP2, RNF182, UPK1B, LRRC31, POSTN, FAM155A, SHISA2) were screened by LASSO (Fig. 6B). A series of 51 genes were obtained after SVM algorithm (Fig. 6C). The 9 feature genes namely NAV3, ITGA10, SYT4, NOX1, SNTG2, RNF182, UPK1B, POSTN, and SHISA2 were yielded after the final screening (Fig. 6D). As seen from the ROC curve of 38 severe asthma samples and 20 healthy samples, the AUC of the 9 feature genes were greater than 0.7 in both GSE43696 dataset and GSE63142 validation set, indicating that the feature genes had a great ability to distinguish severe asthma samples and healthy samples. In the ROC curve of 50 mild to moderate asthma samples and 20 healthy samples, the AUC of 9 feature genes were all over 0.6 in the 2 datesets, suggesting that the feature genes had a good ability to differentiate moderate asthma samples and healthy samples (Fig. 6E). The box plot showed that the expression trends of the feature genes were consistent between asthma and control groups in both datasets (Fig. 6F).

### 3.7. Construction of a nomogram and functional annotation analysis of feature genes

**Figure 7.**
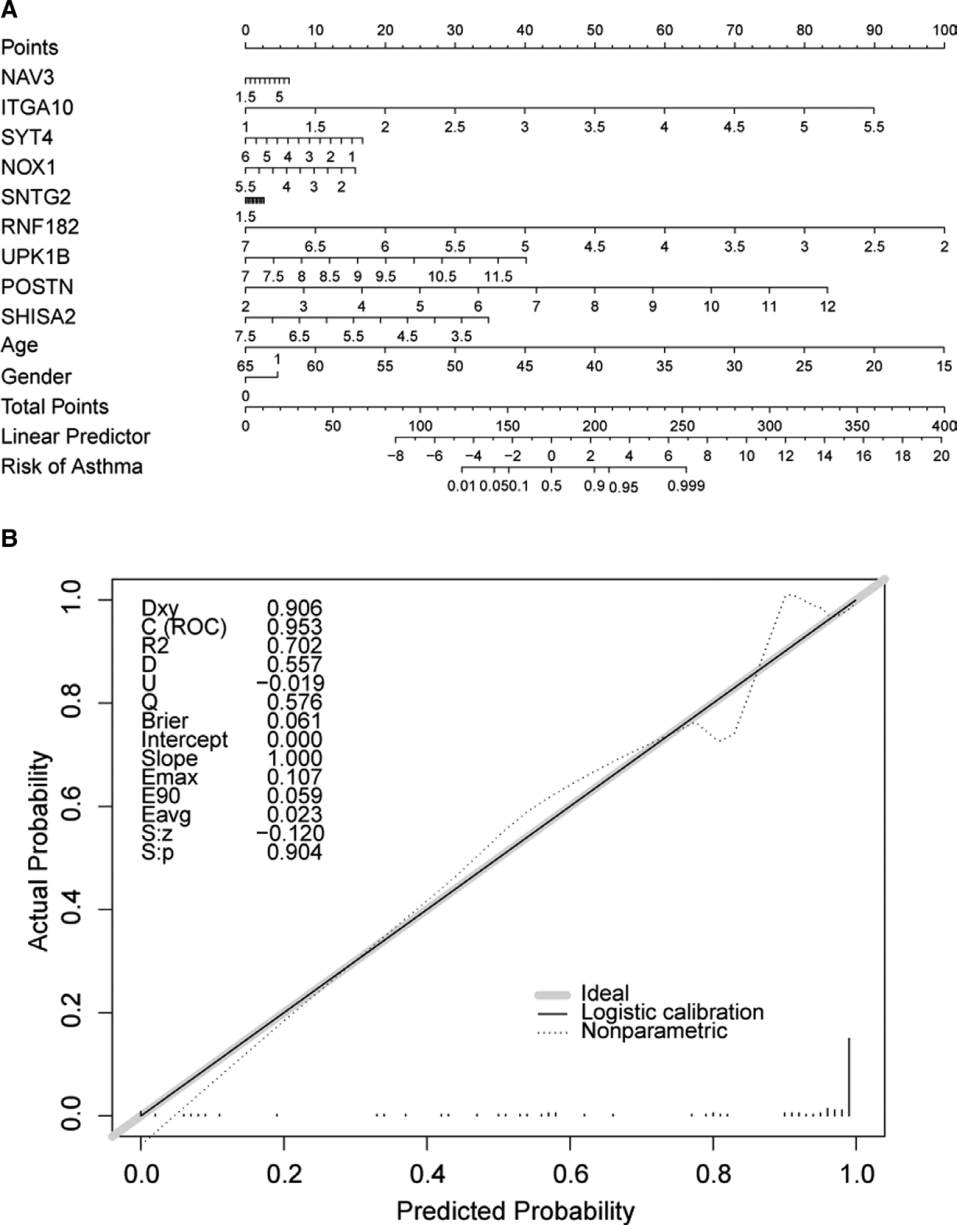
Construction of a nomogram and functional annotation analysis of nine feature genes. (A) Nomogram for predicting probabilities of asthma. For each subjects, eleven lines are drawn upward to determine the value received from the eleven predictors in the nomogram. The sum of these numbers is located on the “Total Points” axis. In addition, a line is drawn downward to determine the possibility of asthma. (B) The calibration curves for predicting the probability of asthma.

9 feature genes (NAV3, ITGA10, SYT4, NOX1, SNTG2, RNF182, UPK1B, POSTN, and SHISA2), age and gender formed the nomogram, and the calibration curve indicated that the prediction ability of nomogram was excellent (Fig. 7A and B). NAV3 was involved in adaptive immune response, cytokine-mediated signaling pathway (GO) and epstein-barr virus infection, herpes simplex virus 1 infection (KEGG). ITGA10 enriched for GO including cytokine-mediated signaling pathway, G protein-coupled receptor activity, etc. and KEGG containing cytokine-cytokine receptor interaction and others. SYT4 enriched for significant GO and KEGG pathways such as cytoplasmic translation, large ribosomal subunit, NOD-like receptor signaling pathway. NOX1 was involved in GO pathways such as endoplasmic reticulum to Golgi vesicle-mediated transport, Golgi vesicle transport, and KEGG signaling pathways contained antigen processing and presentation, intestinal immune network for IgA production, etc. SNTG2 gene enrichment results showed an up-regulation trend for TOP5 GO entries covered chromosome segregation, etc, an up-regulation trend for TOP5 KEGG included proteasome, parkinson disease, huntington disease and a down-regulation trend contained coronavirus disease – COVID-19, herpes simplex virus 1 infection, etc. RNF182 participated in cytoplasmic translation, cytosolic ribosome (GO) and dopaminergic synapse (KEGG) etc. UPK1B enriched in GO and KEGG pathways concluding protein glycosylation (GO), mucin type o-glycan biosynthesis (KEGG) and others. The TOP5 GO entries and KEGG enrichment pathways of POSTN showed an up-regulation trend for cyplasmic translation (GO) and ribosome (KEGG). The GO and KEGG entries that SHISA2 was attended containing DNA-binding transcription activator activity (GO), amino sugar and nucleotide sugar metabolism (KEGG) (see Supplemental Figure S1, Supplemental Digital Content, http://links.lww.com/MD/I556, which demonstrates gene set enrichment analysis for 9 feature genes).

### 3.8. Identification of DE-miRNA and DE-lncRNA and construction of a ceRNA regulatory network

**Figure 8.**
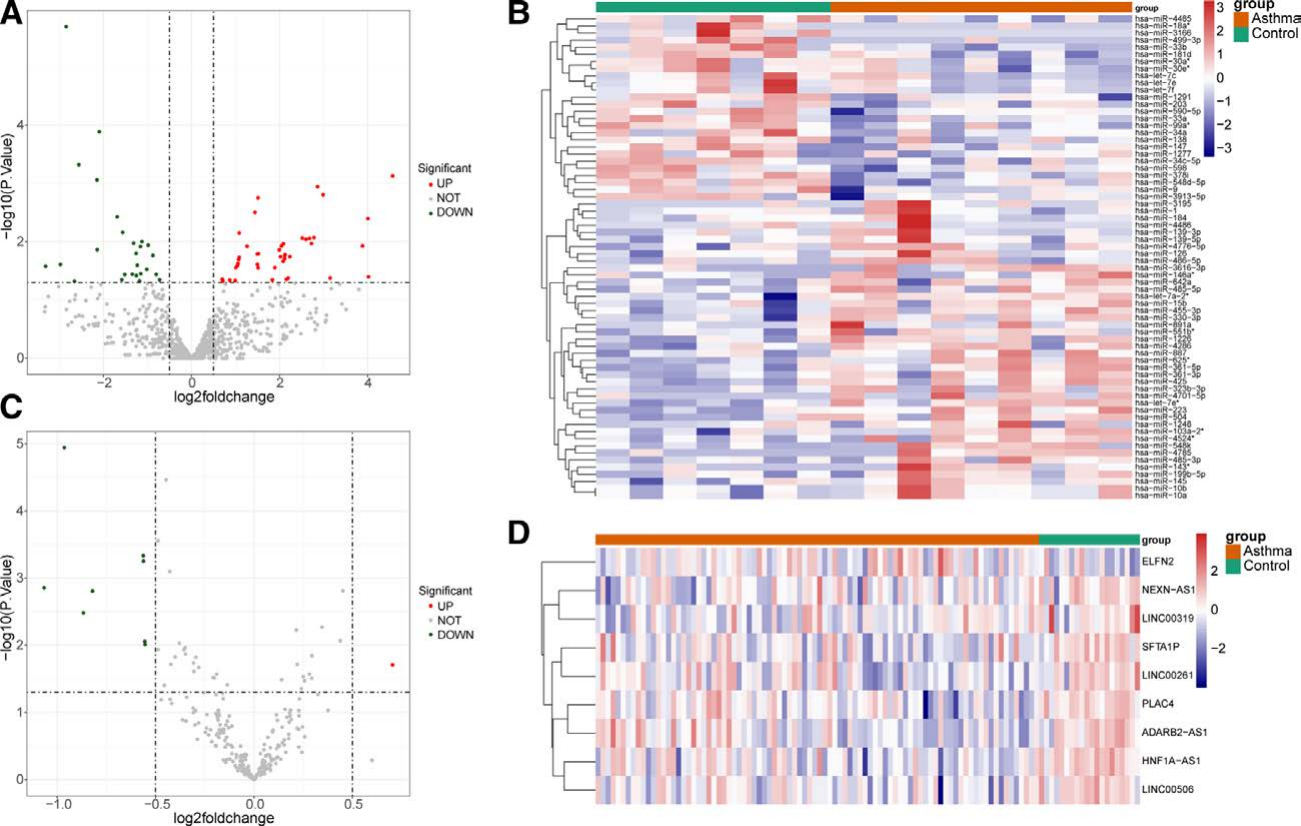
Identification of differentially expressed miRNA and differentially expressed lncRNA. (A) Volcano plot of differentially expressed miRNAs of asthma and normal control subjects. (B) Heat map of up and down differentially expressed miRNAs of asthma and normal control subjects. (C) Volcano plot of differentially expressed lncRNAs of asthma and normal control subjects. (D) Heat map of up and down differentially expressed lncRNAs of asthma and normal control subjects.

**Figure 9.**
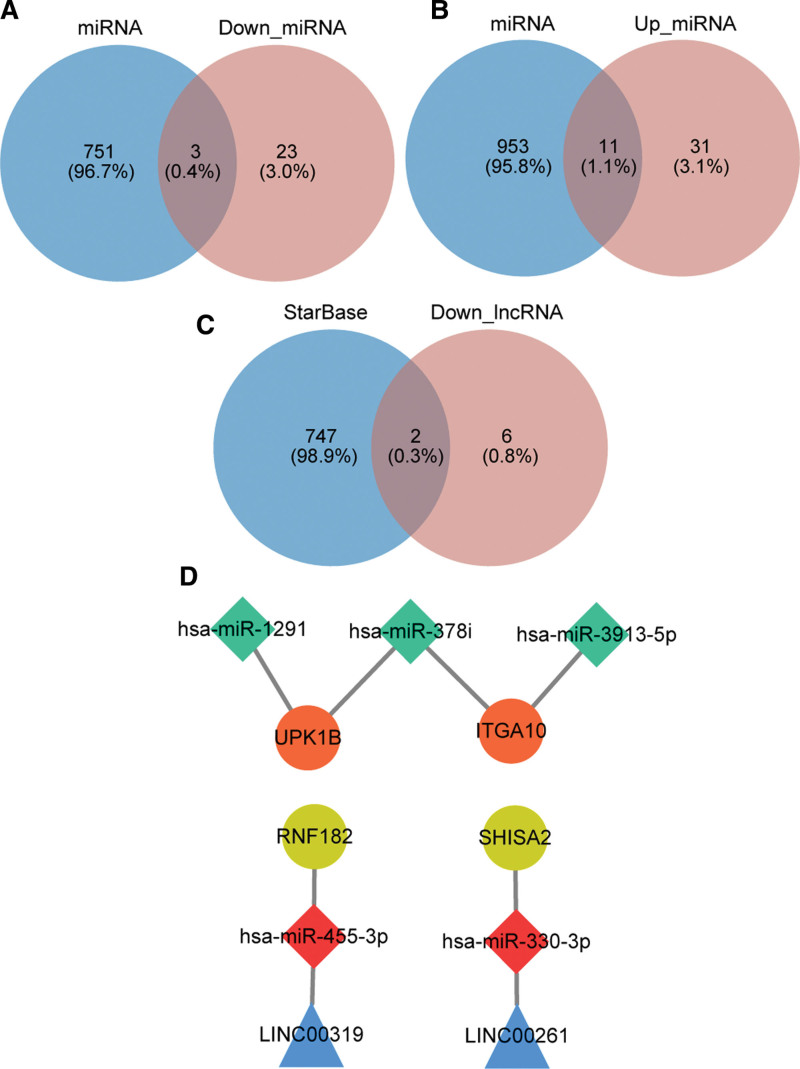
Construction of ceRNA regulatory network. (A) Venn diagram of miRWalk prediction results and down-regulated miRNAs. (B) Venn diagram of miRWalk prediction results and up-regulated miRNAs. (C) Venn diagram of StarBase prediction results and down-regulated lncRNAs. (D) ceRNA network for UPK1B, ITGA10, RNF182, SHISA2. Circles in the figure represent key genes (orange: up-regulated; golden yellow: down-regulated), diamonds represent miRNAs (red: up-regulated; green: down-regulated), triangles represent lncRNAs, and blue represent down-regulated lncRNAs. ceRNA = competing endogenous RNA.

There were 68 DE-miRNAs (42 up-regulated DE-miRNAs and 26 down-regulated DE-miRNAs) and 9 DE-lncRNAs (1 up-regulated lncRNA and 8 down-regulated lncRNAs) between asthma and control comparison groups (Fig. 8A–D). A total of 4 mRNAs, 5 miRNAs and 2 lncRNAs collectively formed a ceRNA regulatory network, and which included RNF182-hsa-miR-455-3p-LINC00319, SHISA2-hsa-miR-330-3p-LINC00261 and other mRNA-miRNA-lncRNA interactions pairs (Fig. 9A–D).

### 3.9. Construction of a mRNA-TF regulatory network

**Figure 10.**
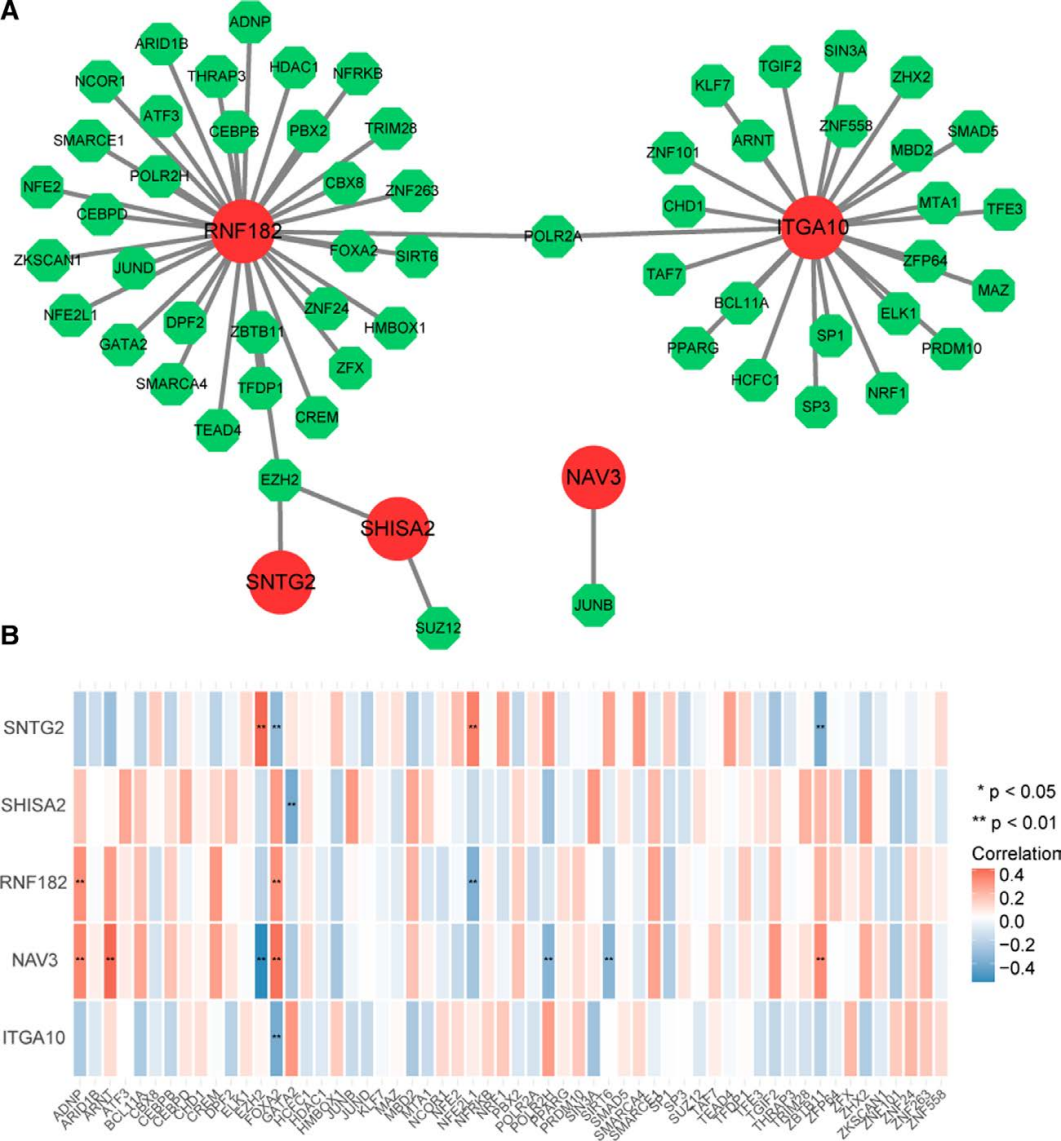
Construction of mRNA-TF regulatory network. (A) mRNA-Tissue factors regulatory network. The red circles in the figure represent key genes, and the green represent tissue factors. (B) Heat map of correlation between key genes and tissue factors. The relationship pairs with asterisks in the figure represent *P* < .05, |cor|>0.3. TF = transcription factor.

A sum of 58 TFs and 5 feature genes (RNF182, ITGA10, SNTG2, SHISA2, NAV3) constituted the mRNA-TF regulatory network which included SNTG2-EZH2, RNF182-FOXA2, and so on (Fig. 10A). The heat map showed that there were differences in the correlations between feature genes and TFs, for example, the SNTG2 was positively a correlated with EZH2, while NAV3 was negatively correlated with EZH2 (Fig. 10B).

### 3.10. Drug sensitivity analysis

**Figure 11.**
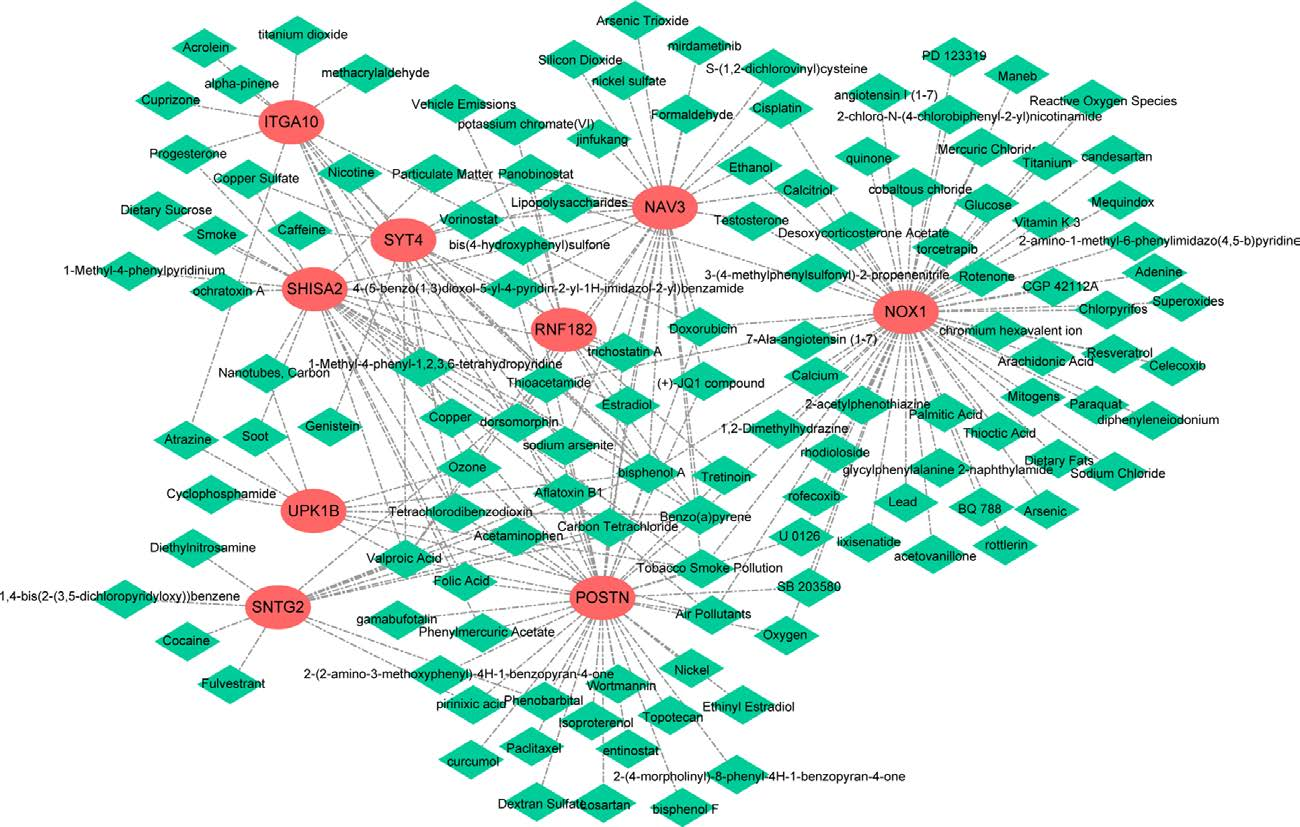
Therapeutic drug network. Network diagram of 9 feature genes and potential therapeutic drugs. Red circles represent 9 feature genes, green diamonds represent predicted therapeutic drugs.

NAV3, ITGA10, SYT4, NOX1, SNTG2, RNF182, UPK1B, POSTN, and SHISA2 were predicted to 28, 14, 15, 60, 13, 10, 11, 41, and 23 drugs in order. The therapeutic drug network diagram included NAV3-bisphenol A, ITGA10-bisphenol A, RNF182-valproic acid and other relationship pairs (Fig. 11, and see Supplemental Table S1, Supplemental Digital Content, http://links.lww.com/MD/I557, which illustrates key genes and predicted potential therapeutics).

## 4. Discussion

This study obtained 438 DEGs in the training set. Then, 9 DE-FRGs were obtained after intersecting with the FerrDb. These 9 DE-FRGs were significantly enriched in BP such as lipoxygenase pathway, response to oxygen levels, and HIF-1 signaling pathway, according to GO and KEGG results. Interestingly, ferroptosis is caused by lipid peroxidation, and lipid metabolism is associated with the onset of asthma. For example, the 15-LO1/PEBP1 complex, which can produce hydroperoxyl-phospholipids, drive ferroptosis and enhance type 2 inflammation-related signaling pathways, is found in the airway epithelial cells of asthmatic patients.^[28]^ In addition, the HIF-1 signaling pathway is the main regulatory pathway for oxygen homeostasis.^[29]^ Under inflammatory conditions, HIF-1α in goblet cells of the airway epithelium can promote the expression of mucin 5AC, which interferes with the mucociliary clearance mechanisms of the airway.^[30,31]^ Additionally, ferroptosis may cause renal tubular damage via the HIF-1α pathway in diabetic kidney disease.^[32]^ Thus, ferroptosis may be involved in the functional changes of asthma airway epithelial cells through multiple ways and may contribute to the development of type 2 inflammation.

After that, we used the STRING database to evaluate the interactions among these 9 DE-FRGs. We obtained 6 DE-FRGs with significant interactions. To verify the ability of these 6 DE-FRGs to discriminate asthma and normal subjects, we performed ROC analysis. According to the AUC value, NOX1 had the best performance (AUC ≥ 0.82), followed by DDIT4, IL-6 and NOS2. NOX1 is an enzyme that produces ROS during ferroptosis and initiates lipid peroxidation. In mouse macrophages and the human lung cancer cell line Calu-1, increased NOX1 leads to lipid peroxidation and subsequent ferroptosis.^[33,34]^ NOX1 has also been mentioned as a marker of moderate to severe asthma. It is involved in the ion transport regulatory pathway and oxidative stress in airway epithelial cells of asthma.^[35–37]^ DDIT4 (also known as Rtp801) is a developmental glucocorticoid gene, and may be involved in the susceptibility to asthma and lung development.^[38]^ DDIT4 can inhibit the mTOR signaling in mouse fibroblasts, it is necessary for amplifying oxidative stress caused by cigarette smoke.^[39]^ IL-6 also can promote lipid peroxidation and induce ferroptosis, which can be reversed by Fer-1 treatment.^[40]^ NOS2, a nitric oxide synthase gene, is strongly correlated with the levels of fractional exhaled nitric oxide which is a marker of airway inflammation in asthma.^[41,42]^ And ferroptosis can induce the expression of NOS2 in macrophages, Th2 cytokines can induce the expression of NOS2 either.^[43]^ In some studies,^[44]^ MEF2C knockout enhanced the effect of ferroptosis inducer Erastin on meningioma cell ferroptosis and lipid peroxidation in vitro, and strengthened the meningioma growth inhibition mediated by ferroptosis in mouse models, this may be related to the downregulation of NF2 and E-cadherin. MEF2C is also proposed as a biomarker for asthma, which decreases with the increase of asthma severity.^[45]^ The number increase of airway blood vessels is a feature of airway remodeling in asthma. It is shown that the levels of VEGFA in alveolar lavage fluid in asthmatic patients correlate with the number of blood vessels.^[46]^ Further more, ferroptosis in endometrial stromal cells triggers the production of VEGFA and IL-8, thus promoting angiogenesis in adjacent lesions and accelerating disease progression.^[47]^ These findings suggest that these 6 interactions genes may play a vital role in ferroptosis in asthma. Whether they can jointly promote the ferroptosis in airway epithelial cells, and how they interact, needs to be further studied.

Asthma is a heterogeneous disease^.[48]^ To further analyze the function of ferroptosis in airway epithelial cells, we divided asthmatic patients into two subtypes according to 9 DE-FRGs. Through GSVA analysis, we found that there were obvious differences in biological functions between the two subtypes. And DEGs between the two subtypes were significantly enriched in the inflammatory signaling pathways, such as cytokine-cytokine receptor interaction. In IL-13 stimulated bronchial epithelial cell line (BEAS-2B), Fer-1 treatment reduced ferroptosis and oxidative damage, and inhibited the production of inflammatory cytokines.^[49]^ In another study,^[28]^ IL-13 induced the production of hydroperoxyl-phospholipids by 15-LO1, reduced intracellular GSH, and increased extracellular oxidative GSH. This redox imbalance makes airway epithelial cells more sensitive to ferroptosis. Cellular GSH is further reduced by inhibition of SLC7A11, promoting ferroptosis and expression of type 2 inflammatory cytokines like CCL26 and POSTN. Moreover, IL-13 induced mucin 5AC is regulated by the 15-LO1 pathway in human bronchial epithelial cells, indicating that inflammatory cytokines-induced mucus secretion is regulated by the ferroptosis-related pathway.^[50]^ In sum, ferroptosis and inflammatory cytokines promote each other and interfere with the function of airway epithelial cells. In this study, we obtained consistent results, demonstrating that the inflammatory response is related to ferroptosis in the asthmatic airway epithelium. A variety of inflammatory cells and cytokines participate in asthma, which inflammatory cytokines (such as IL-13) could cause ferroptosis of airway epithelial cells and which ones do not, a further study is required.

To further narrow down the genes significantly associated with ferroptosis in airway epithelial cells in asthmatic patients, we intersected DEGs between asthma and control samples, inter-cluster DEGs and asthma-related module. We obtained 88 asthma-related ferroptosis associated genes. Then, LASSO and SVM-RFE were used to identify the critical ferroptosis associated genes involved in asthma. Finally, 9 feature genes (NAV3, ITGA10, SYT4, NOX1, SNTG2, RNF182, UPK1B, POSTN, and SHISA2) were identified as the essential genes. Next, we also analyzed the ability of these 9 feature genes to distinguish between asthma and normal subjects. Our results showed that these 9 feature genes could clearly distinguish between normal and mild to moderate asthma, as well as between normal and severe asthma in two datasets. As we can see, they had better diagnostic ability for severe asthma, this indicated that ferroptosis may be associated with asthma severity. Among these 9 feature genes, RNF182 had the best ability in distinguishing severe asthma, with AUC of 0.889 (GSE43696) and 0.849 (GSE63142), respectively. But NOX1 had the best ability to differentiate between mild and moderate asthma, with AUC of 0.798 (GSE43696) and 0.826 (GSE63142), respectively. This indicates that there are specific differences in airway epithelial cell physiological functions between mild to moderate asthmatic patients and severe asthmatic patients. Notably, NOX1 was screened from the ferroptosis database and our subsequent machine learning analysis, demonstrating its importance in ferroptosis of asthma airway epithelial cells. To evaluate the comprehensive diagnostic ability of these 9 feature genes for asthma, we performed nomogram analysis. The results showed that these 9 feature genes had good discrimination ability for asthma, with age, RNF182 and ITGA10 being the top three contributing factors. However, in addition to age and gender, more clinical indicators need to be added in the future.

After GSEA analysis, we found that POSTN-associated genes were involved in adaptive immune responses; NOX1-associated genes were involved in antigen processing and presentation; RNF182-associated genes were involved in viral infection and innate immunodeficiency; and, SYT4-associated genes were involved in the NOD-like receptor signaling pathway and TGF-β signaling pathway. They are all associated with immune response, may be crucial in the development of asthma. It has been shown that POSTN upregulation in vascular smooth muscle cells can increase cell sensitivity to ferroptosis by inhibiting SLC7A11 expression, suppressing P53, and reducing GSH synthesis.^[51]^ POSTN is also thought to be involved in airway remodeling of asthma,^[52]^ and IL4 and IL13 can promote the expression of POSTN in airway epithelial cells. Furthermore, POSTN is considered as a marker of Th2-high asthma.^[53]^ RNF182 increased expression in hepatocellular carcinoma can mediate p65 ubiquitination, thus accelerating the degradation of p65 protein, blocking the binding of p65 to the SLC7A11 promoter, and promoting ferroptosis.^[54]^ In the LNCaP prostate cancer cell line, the ferroptosis inducer Erastin can increase SYT4 expression. In contrast, the ferroptosis inhibitor Fer-1 can reduce SYT4 expression, suggesting that SYT4 is associated with ferroptosis.^[55]^ However, the mechanisms underlying the role of RNF182 and SYT4 in ferroptosis of asthmatic airway epithelial cells have not been reported. UPK1B is significantly associated with DNA methylation sites in whole blood of asthma patients, but the biological mechanism is unclear.^[56]^ SHISA2, SNTG2, NAV3, and ITGA10 are new genes that have not been previously reported in ferroptosis and asthma, and their role in ferroptosis of asthmatic airway epithelial cells remains to be explored. In summary, there are few studies on the ferroptosis mechanism of these 9 feature genes in asthmatic airway epithelial cells. Fortunately, we found miRNAs, lncRNAs, transcription factors, and therapeutic drug associated with these genes in the network analysis, which may provide reference for mechanistic study of ferroptosis.

## 5. Conclusion

This study firstly found that the ferroptosis genes could distinguish asthma patients from normal subjects. Secondly, through the methods of WGCNA and machine learning, 9 feature genes (NAV3, ITGA10, SYT4, NOX1, SNTG2, RNF182, UPK1B, POSTN, SHISA2) related to ferroptosis were identified. The diagnostic ability of these 9 feature genes for asthma was verified. The regulatory network and intervention drugs were analyzed. However, the causal relationship between ferroptosis and these 9 feature genes could not be determined, which is one of the limitations of this study. In the future, we will continue to investigate the specific molecular mechanisms of these 9 feature genes in ferroptosis of airway epithelial cells, so as to provide theoretical basis for understanding asthma and ferroptosis of airway epithelial cells.

## Acknowledgments

We would like to thank TopEdit (www.topeditsci.com) for its linguistic assistance during the preparation of this manuscript.

## Author contributions

**Conceptualization:** Ye Zheng, Xiaofeng Jiang.

Data curation: Ye Zheng.

Formal analysis: Ye Zheng.

Investigation: Ye Zheng.

Methodology: Ye Zheng.

Project administration: Ye Zheng.

Resources: Ye Zheng.

Software: Ye Zheng.

Supervision: Jingyao Fan, Xiaofeng Jiang.

Validation: Ye Zheng, Jingyao Fan.

Visualization: Ye Zheng.

Writing – original draft: Ye Zheng.

Writing – review & editing: Jingyao Fan, Xiaofeng Jiang.

## Supplementary Material




