# Supplementary material for: The role of ferroptosis-related genes in airway epithelial cells of asthmatic patients based on bioinformatics
Source: Medicine (Baltimore). 2023 Mar 3;102(9):e33119. doi: 10.1097/MD.0000000000033119 (PMC9981416; doi:10.1097/MD.0000000000033119)
Supplement: Supplementary file 1 [file medi-102-e33119-s001.pdf]

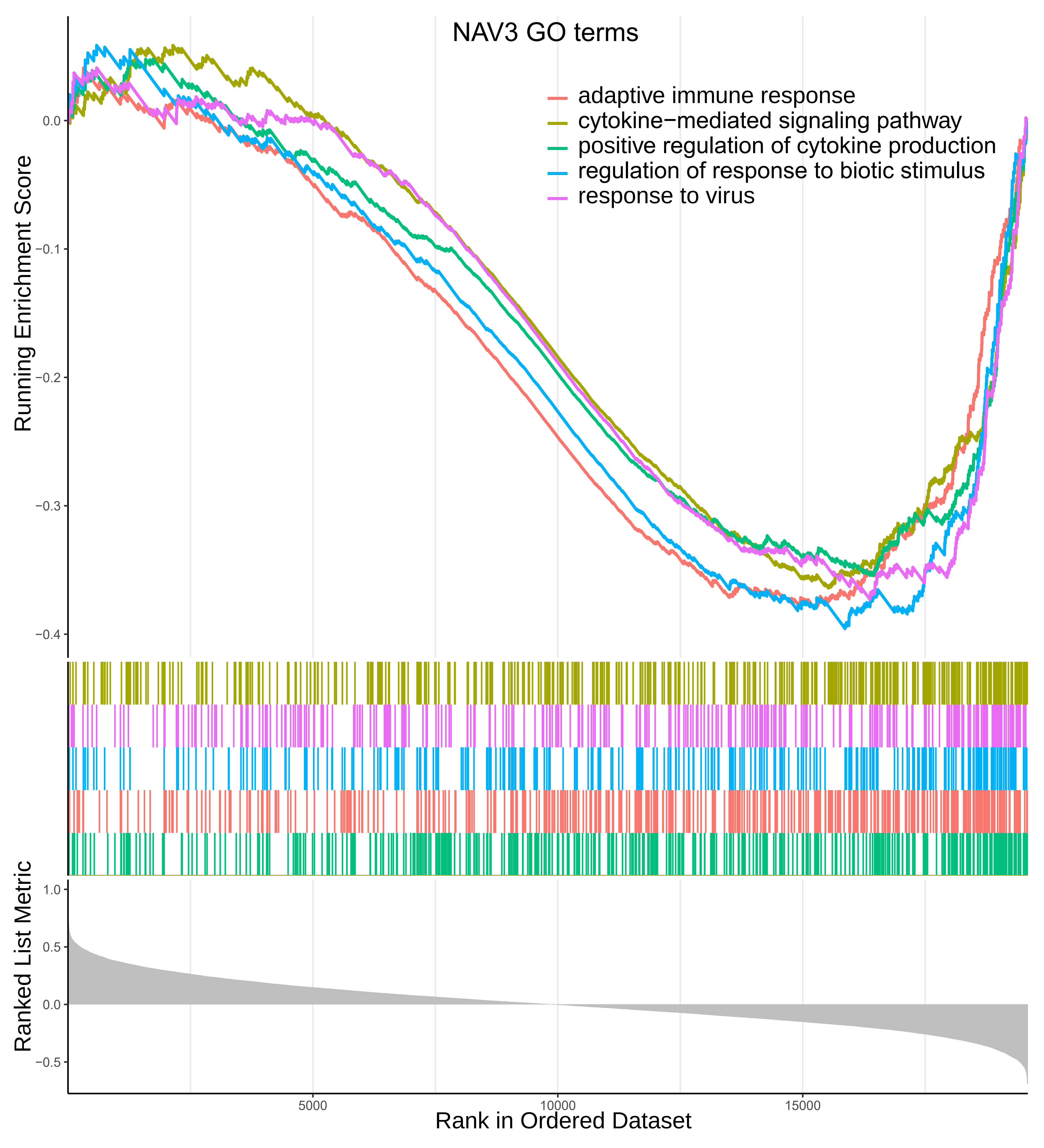
NAV3 GO terms
adaptive immune response
cytokine−mediated signaling pathway
positive regulation of cytokine production
regulation of response to biotic stimulus
response to virus
Running Enrichment Score
0.0
−0.1
−0.2
−0.3
−0.4
Ranked List Metric
1.0
0.5
0.0
−0.5
5000
10000
15000
Rank in Ordered Dataset

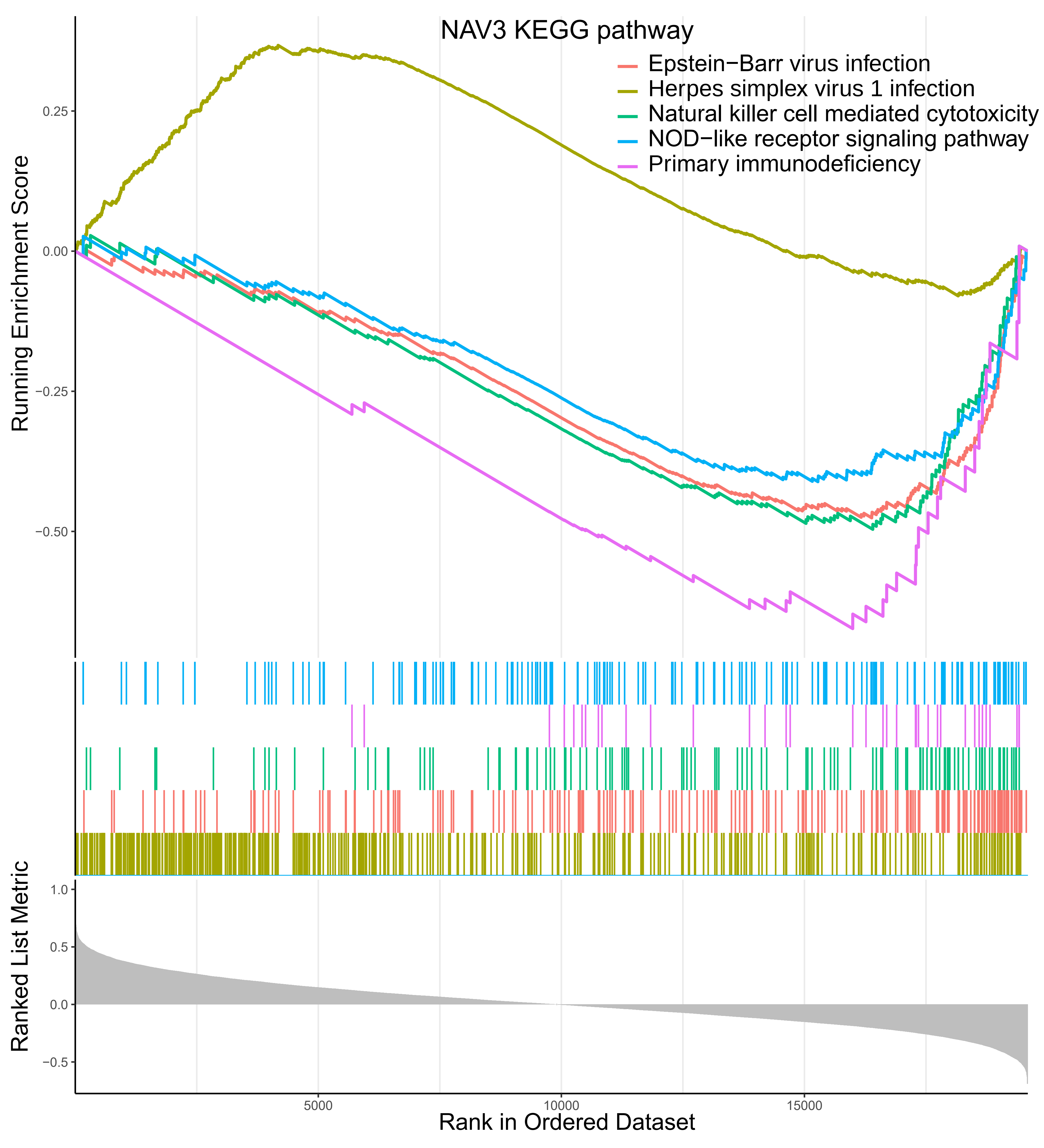
NAV3 KEGG pathway
Epstein−Barr virus infection
Herpes simplex virus 1 infection
Natural killer cell mediated cytotoxicity
NOD−like receptor signaling pathway
Primary immunodeficiency
Running Enrichment Score
0.25
0.00
−0.25
−0.50
Ranked List Metric
1.0
0.5
0.0
−0.5
5000
10000
15000
Rank in Ordered Dataset

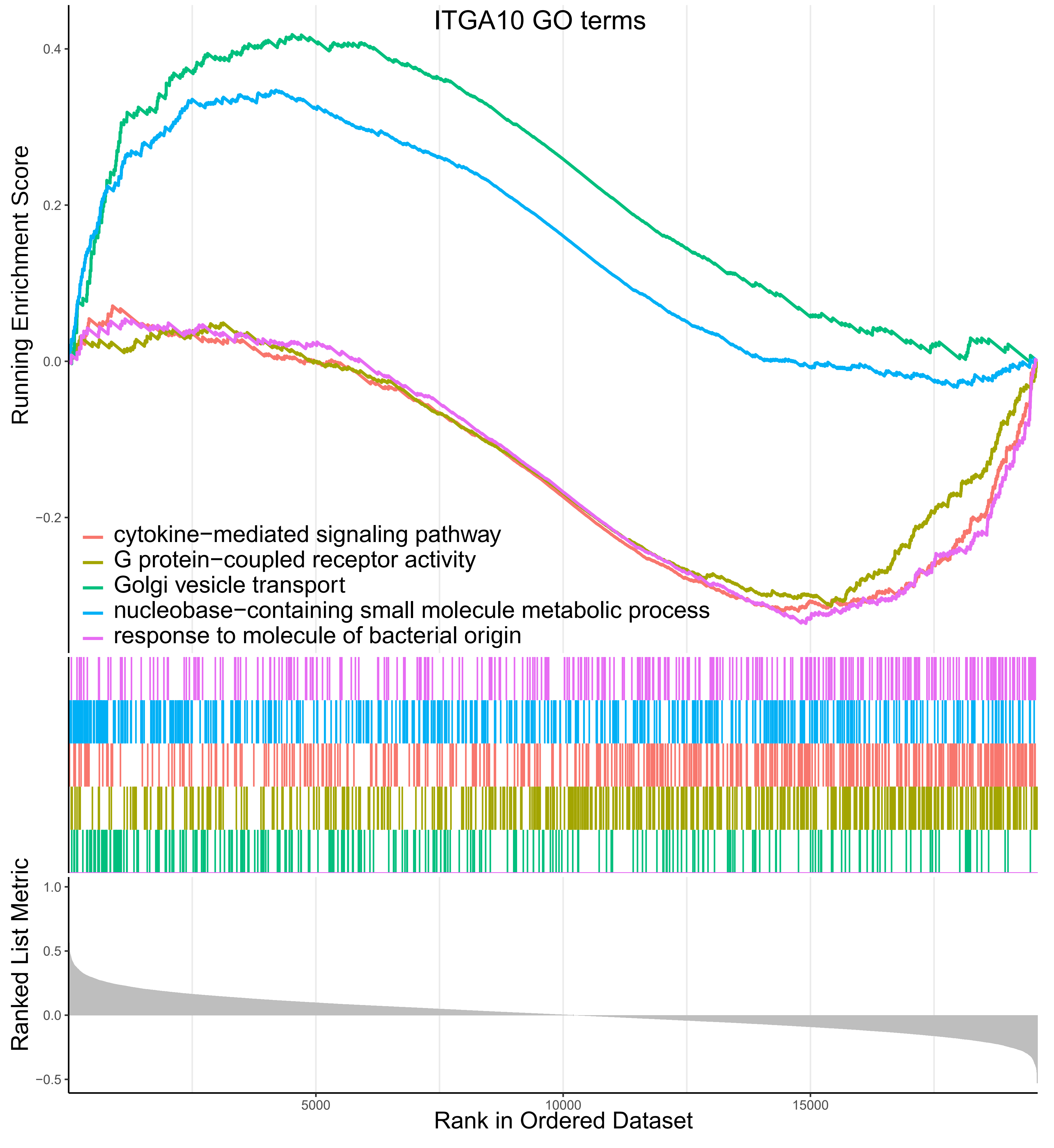

ITGA10 GO terms
Running Enrichment Score
0.4
0.2
0.0
−0.2
cytokine−mediated signaling pathway
G protein−coupled receptor activity
Golgi vesicle transport
nucleobase−containing small molecule metabolic process
response to molecule of bacterial origin
Ranked List Metric
1.0
0.5
0.0
−0.5
5000
10000
15000
Rank in Ordered Dataset

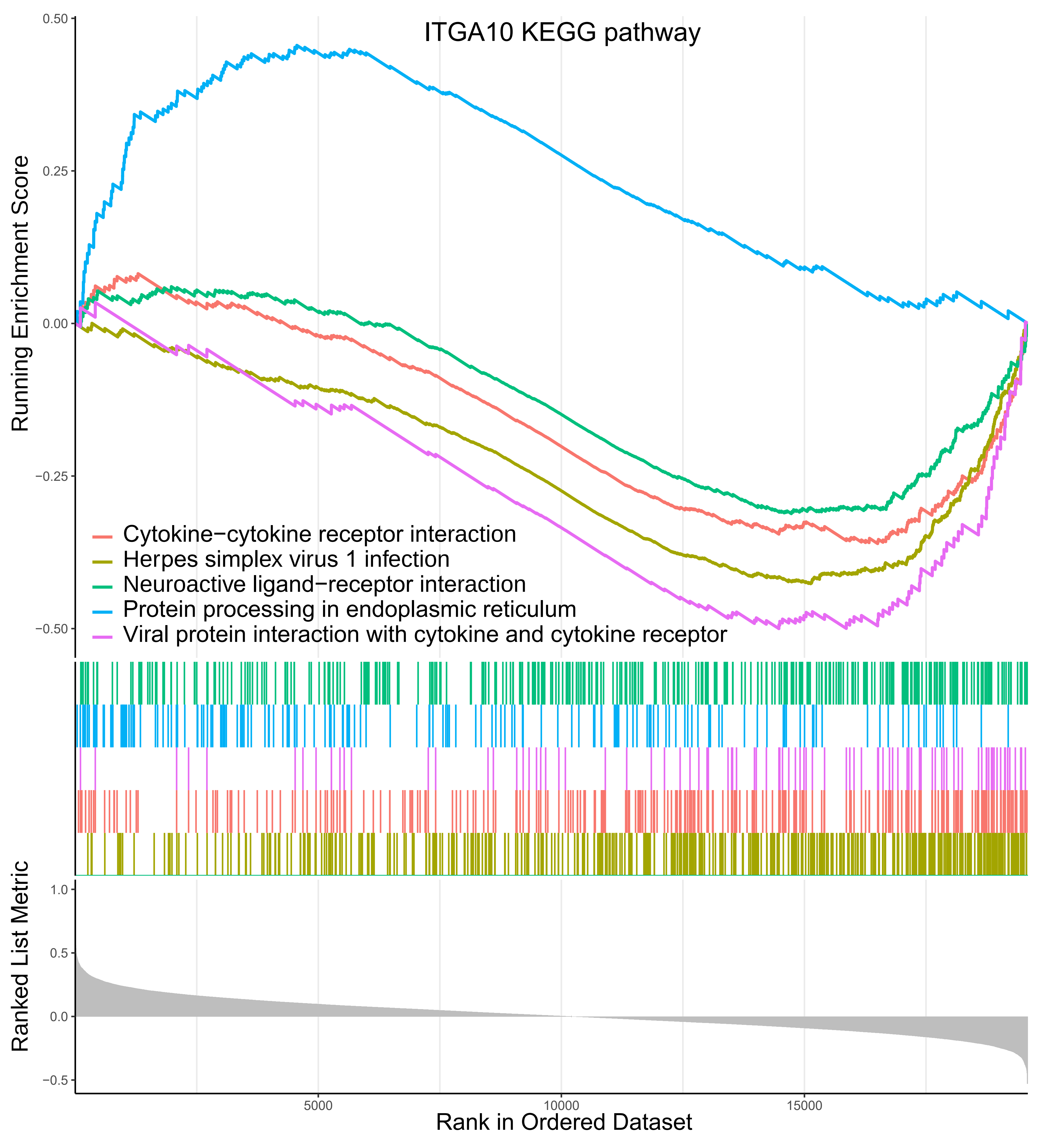

ITGA10 KEGG pathway
Running Enrichment Score
0.50
0.25
0.00
−0.25
−0.50
Cytokine−cytokine receptor interaction
Herpes simplex virus 1 infection
Neuroactive ligand−receptor interaction
Protein processing in endoplasmic reticulum
Viral protein interaction with cytokine and cytokine receptor
Ranked List Metric
1.0
0.5
0.0
−0.5
5000
10000
15000
Rank in Ordered Dataset

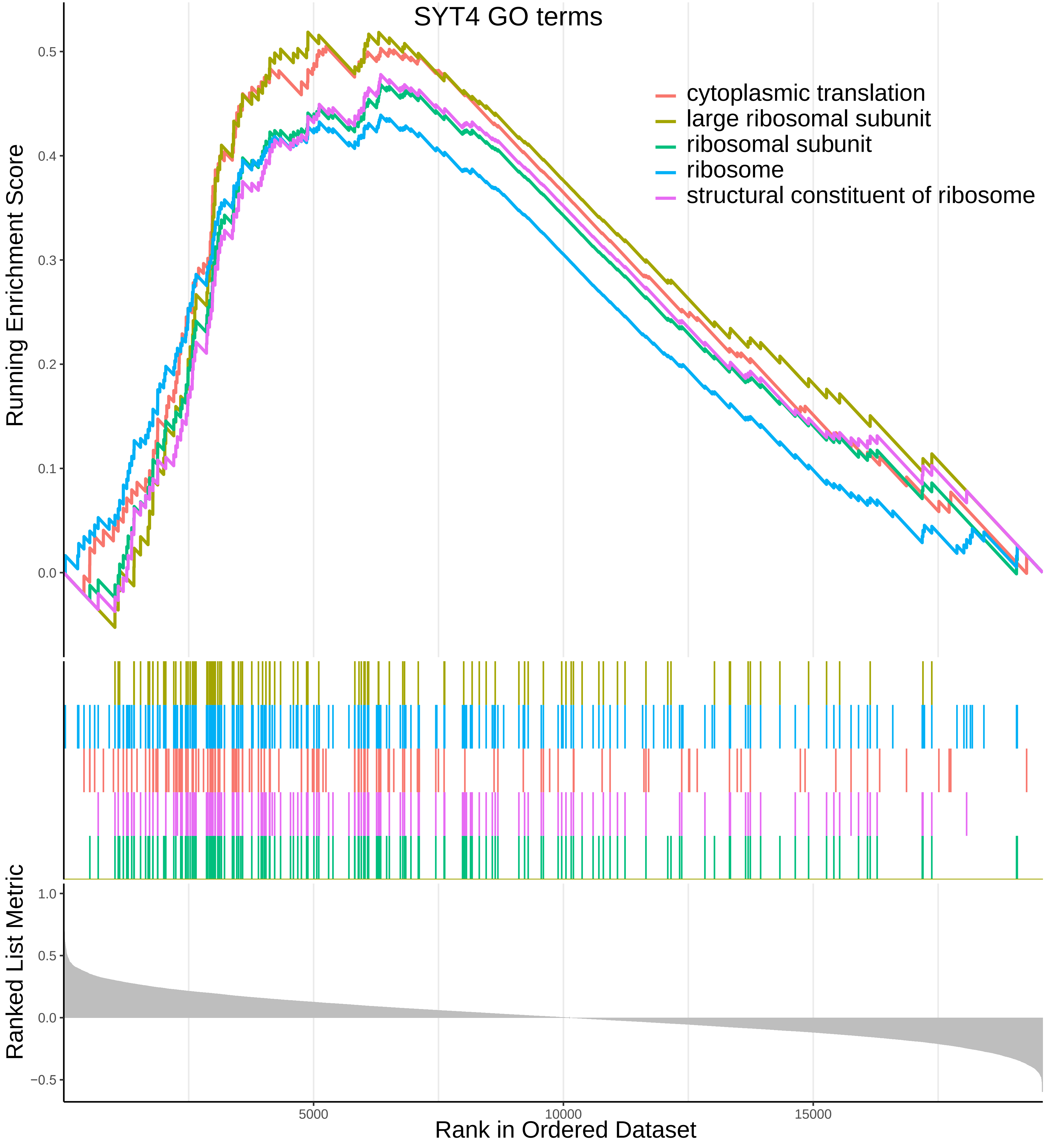
SYT4 GO terms
Running Enrichment Score
0.5
0.4
0.3
0.2
0.1
0.0
cytoplasmic translation
large ribosomal subunit
ribosomal subunit
ribosome
structural constituent of ribosome
Ranked List Metric
1.0
0.5
0.0
−0.5
5000
10000
15000
Rank in Ordered Dataset

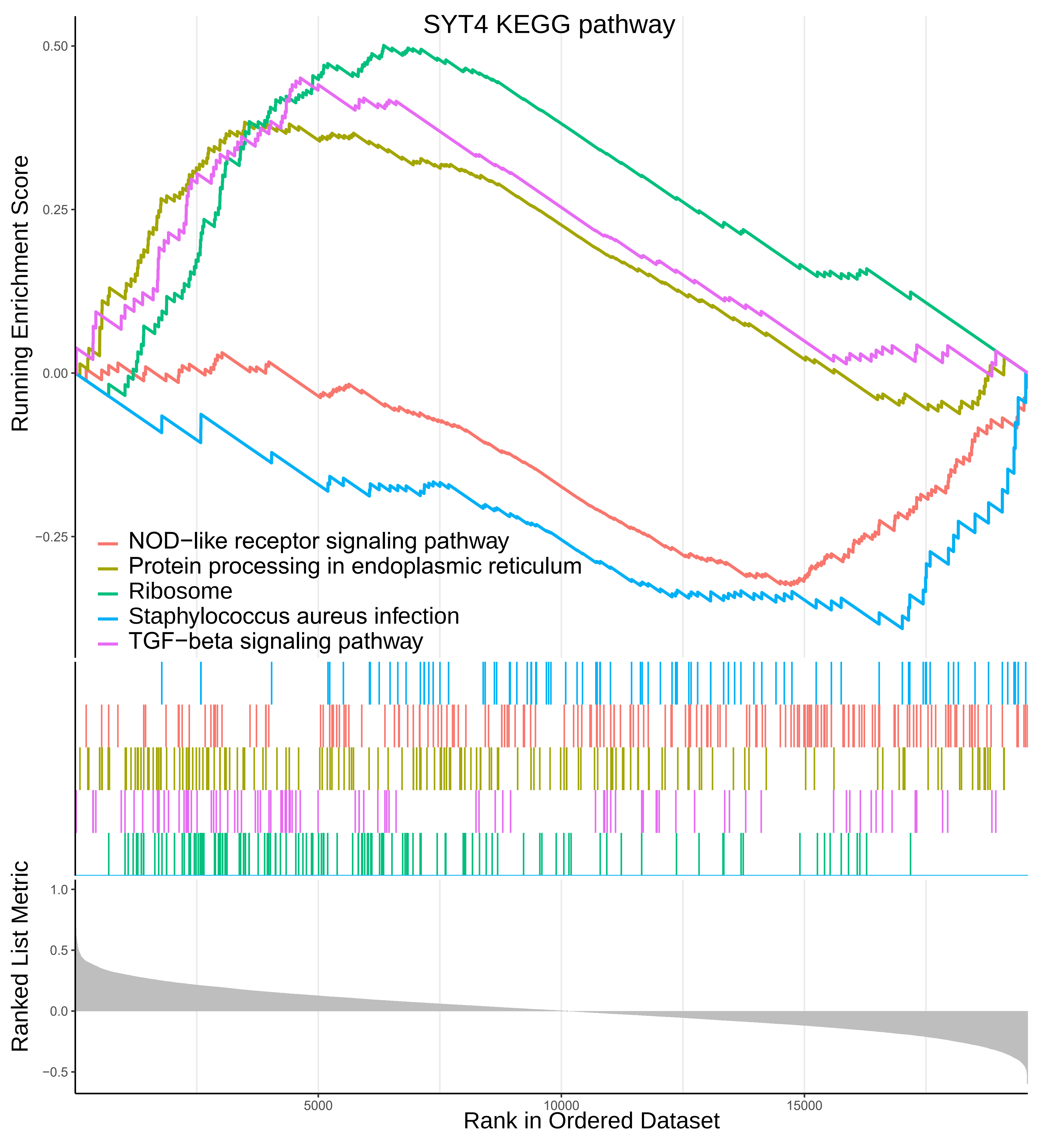
SYT4 KEGG pathway
Running Enrichment Score
0.50
0.25
0.00
−0.25
NOD−like receptor signaling pathway
Protein processing in endoplasmic reticulum
Ribosome
Staphylococcus aureus infection
TGF−beta signaling pathway
Ranked List Metric
1.0
0.5
0.0
−0.5
5000
10000
15000
Rank in Ordered Dataset

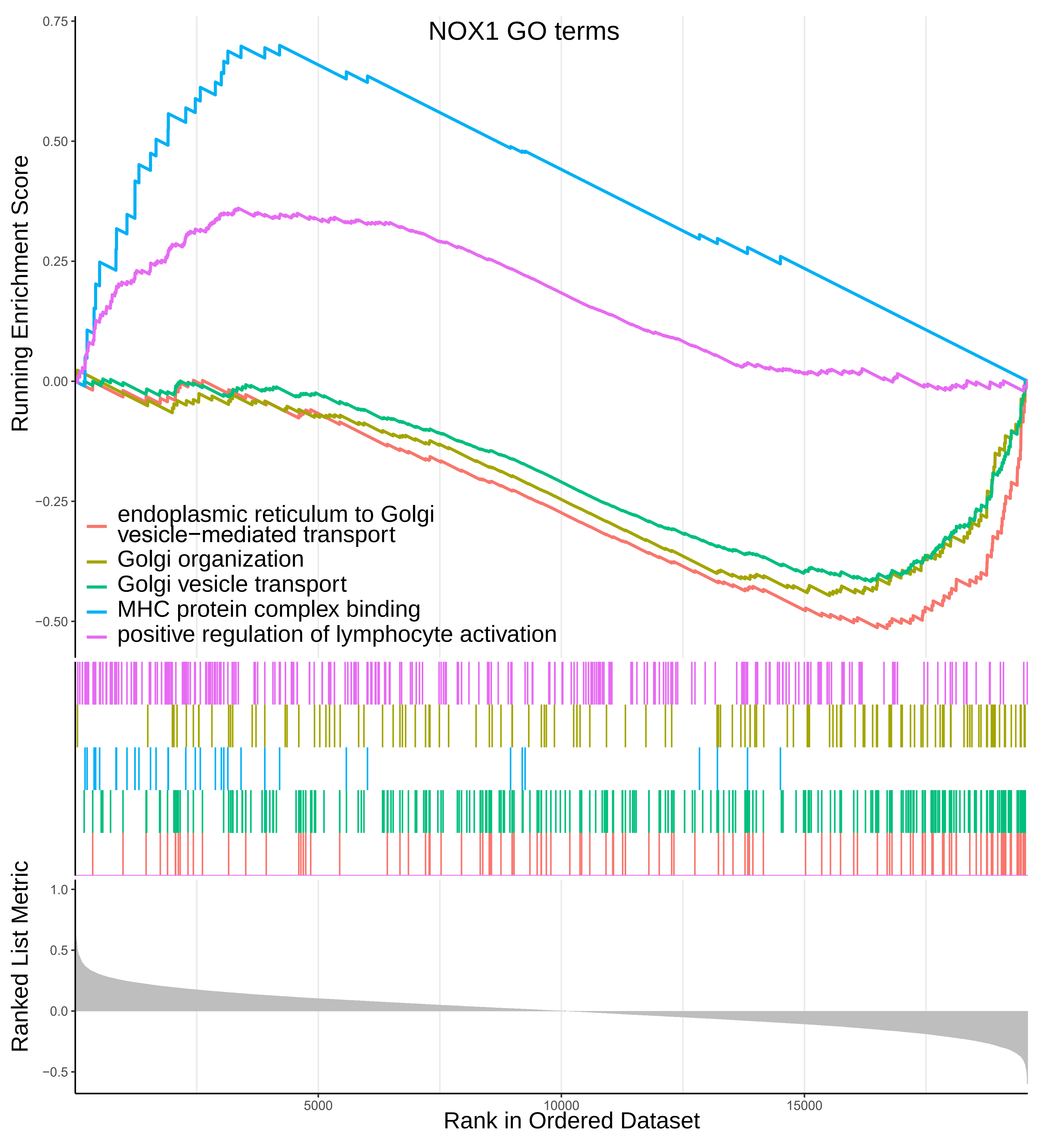
NOX1 GO terms
Running Enrichment Score
0.75
0.50
0.25
0.00
−0.25
−0.50
endoplasmic reticulum to Golgi vesicle−mediated transport
Golgi organization
Golgi vesicle transport
MHC protein complex binding
positive regulation of lymphocyte activation
Ranked List Metric
1.0
0.5
0.0
−0.5
5000
10000
15000
Rank in Ordered Dataset

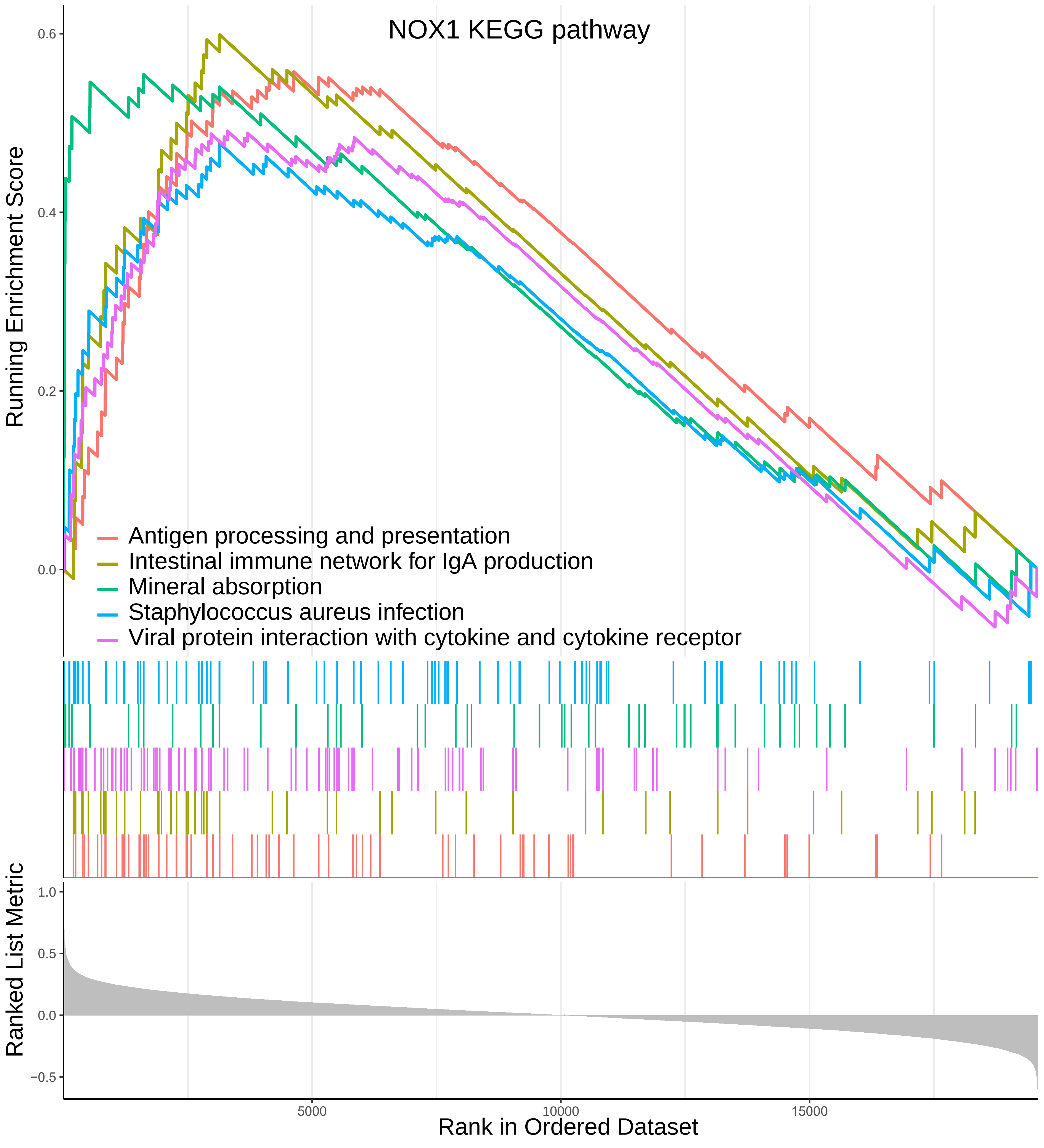

NOX1 KEGG pathway
Running Enrichment Score
0.6
0.4
0.2
0.0
Antigen processing and presentation
Intestinal immune network for IgA production
Mineral absorption
Staphylococcus aureus infection
Viral protein interaction with cytokine and cytokine receptor
Ranked List Metric
1.0
0.5
0.0
−0.5
5000
10000
15000
Rank in Ordered Dataset

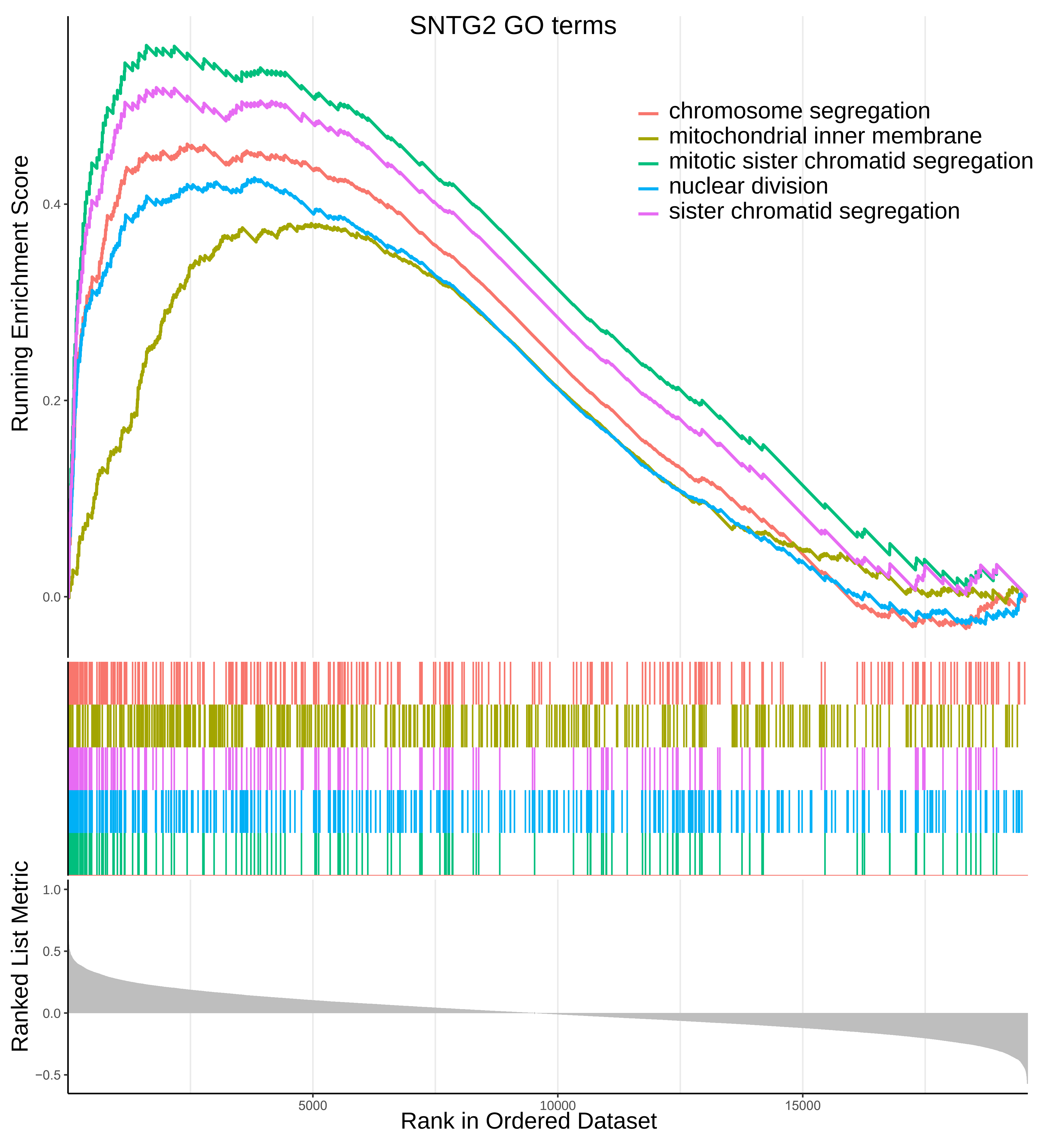
SNTG2 GO terms
chromosome segregation
mitochondrial inner membrane
mitotic sister chromatid segregation
nuclear division
sister chromatid segregation
Running Enrichment Score
0.4
0.2
0.0
Ranked List Metric
1.0
0.5
0.0
−0.5
5000
10000
15000
Rank in Ordered Dataset

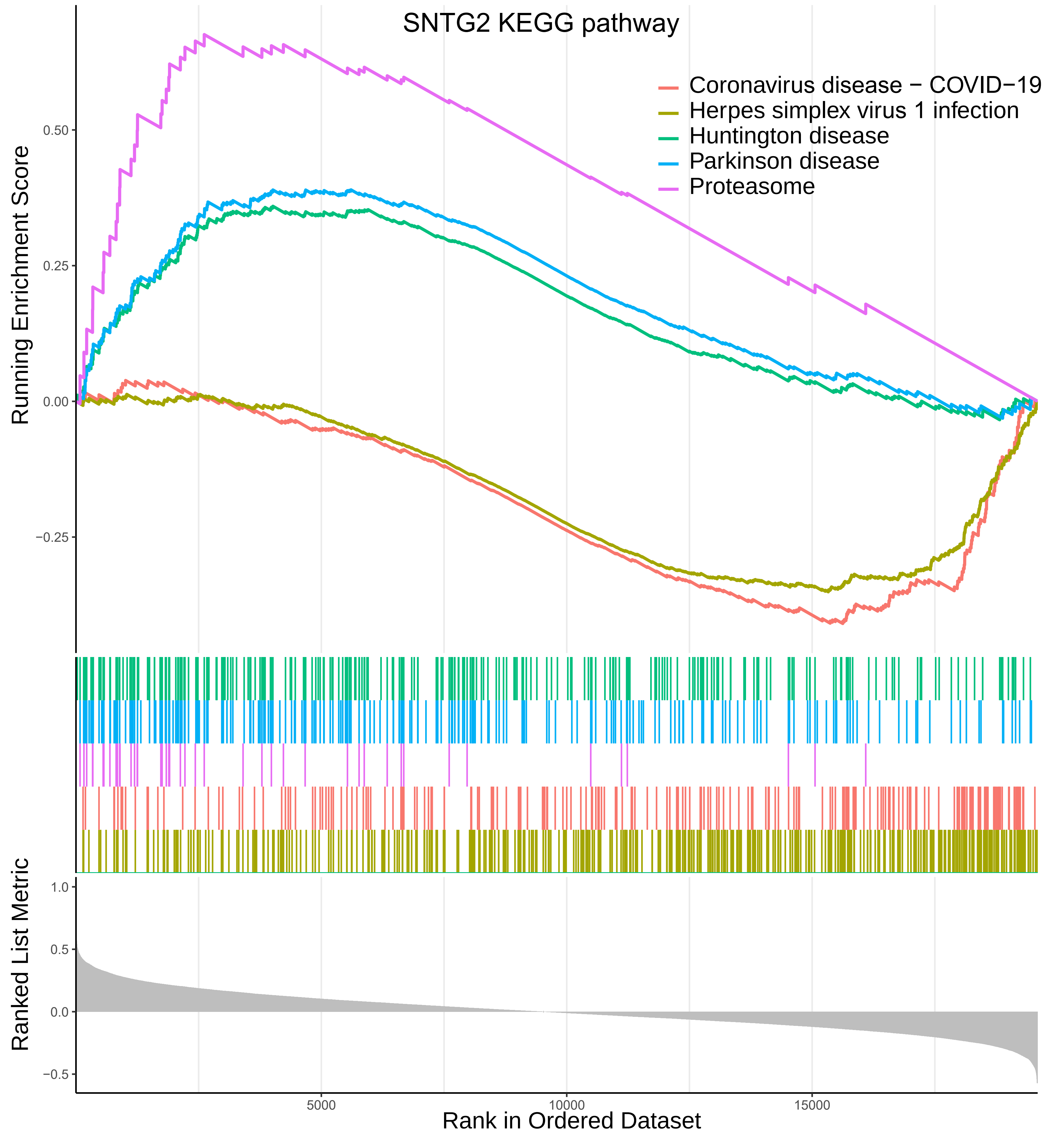

SNTG2 KEGG pathway
Coronavirus disease − COVID−19
Herpes simplex virus 1 infection
Huntington disease
Parkinson disease
Proteasome
Running Enrichment Score
0.50
0.25
0.00
−0.25
Ranked List Metric
1.0
0.5
0.0
−0.5
5000
10000
15000
Rank in Ordered Dataset

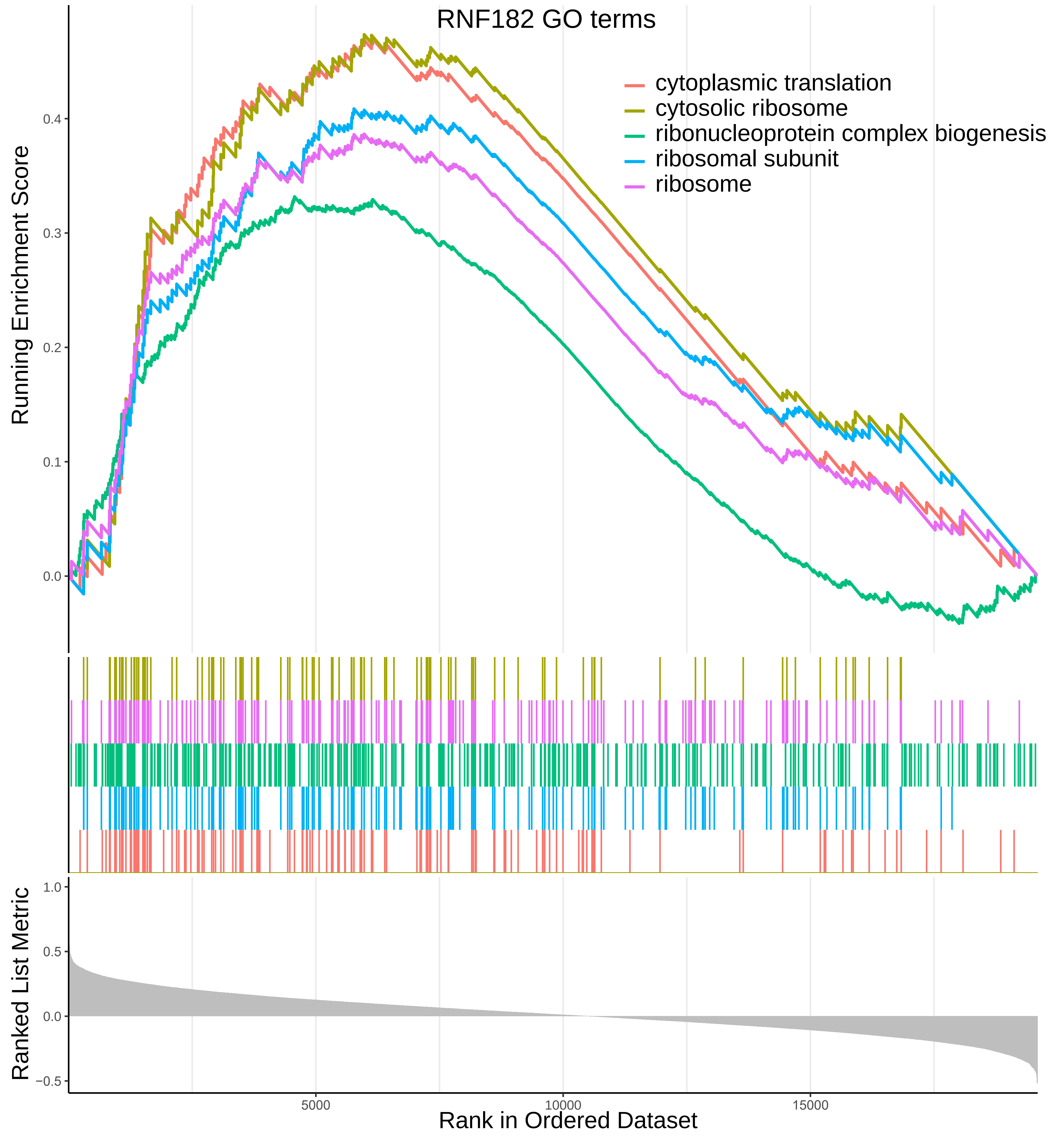

RNF182 GO terms
cytoplasmic translation
cytosolic ribosome
ribonucleoprotein complex biogenesis
ribosomal subunit
ribosome
Running Enrichment Score
0.4
0.3
0.2
0.1
0.0
Ranked List Metric
1.0
0.5
0.0
−0.5
5000
10000
15000
Rank in Ordered Dataset

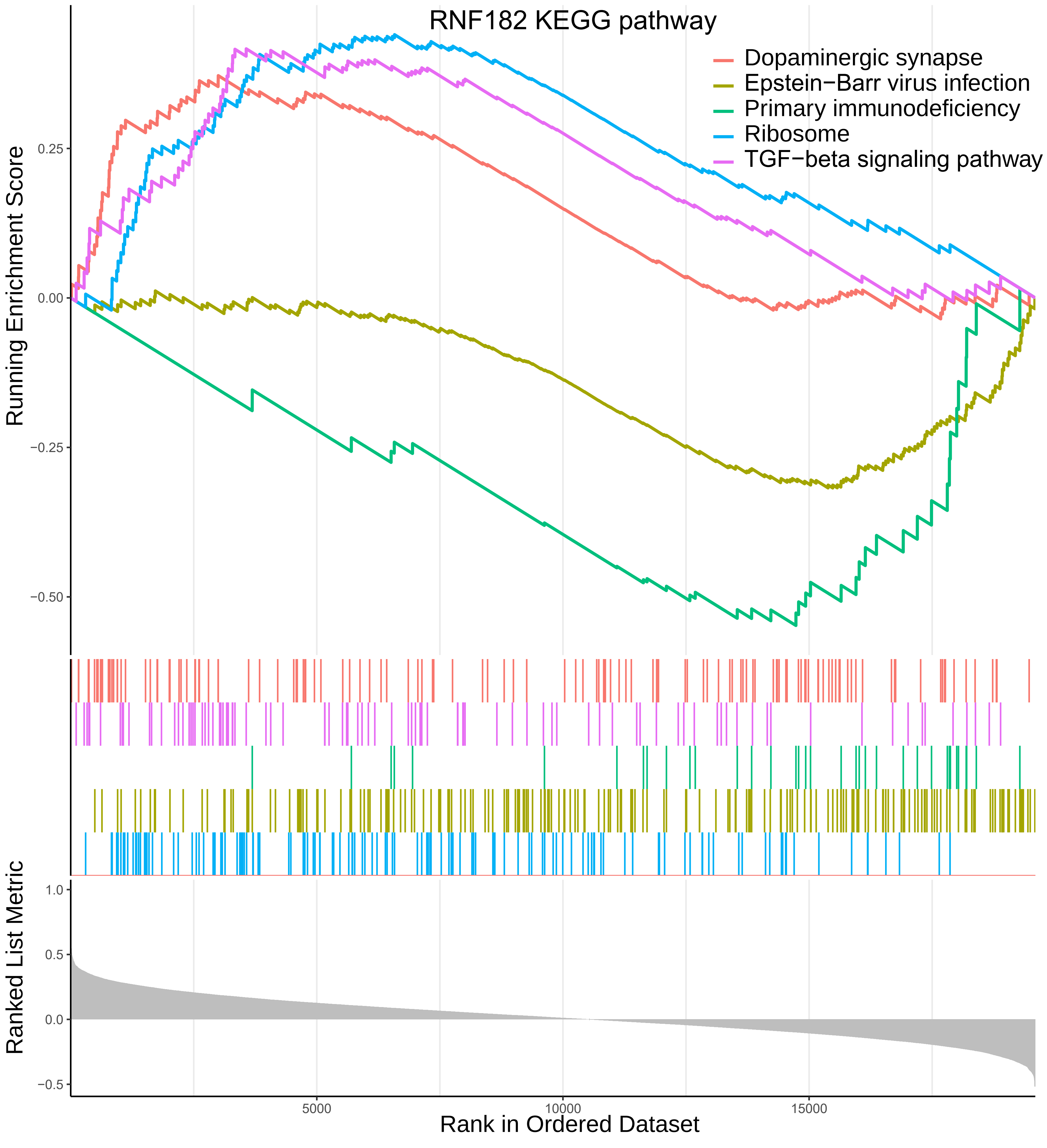
RNF182 KEGG pathway
Dopaminergic synapse
Epstein−Barr virus infection
Primary immunodeficiency
Ribosome
TGF−beta signaling pathway
Running Enrichment Score
0.25
0.00
−0.25
−0.50
Ranked List Metric
1.0
0.5
0.0
−0.5
5000
10000
15000
Rank in Ordered Dataset

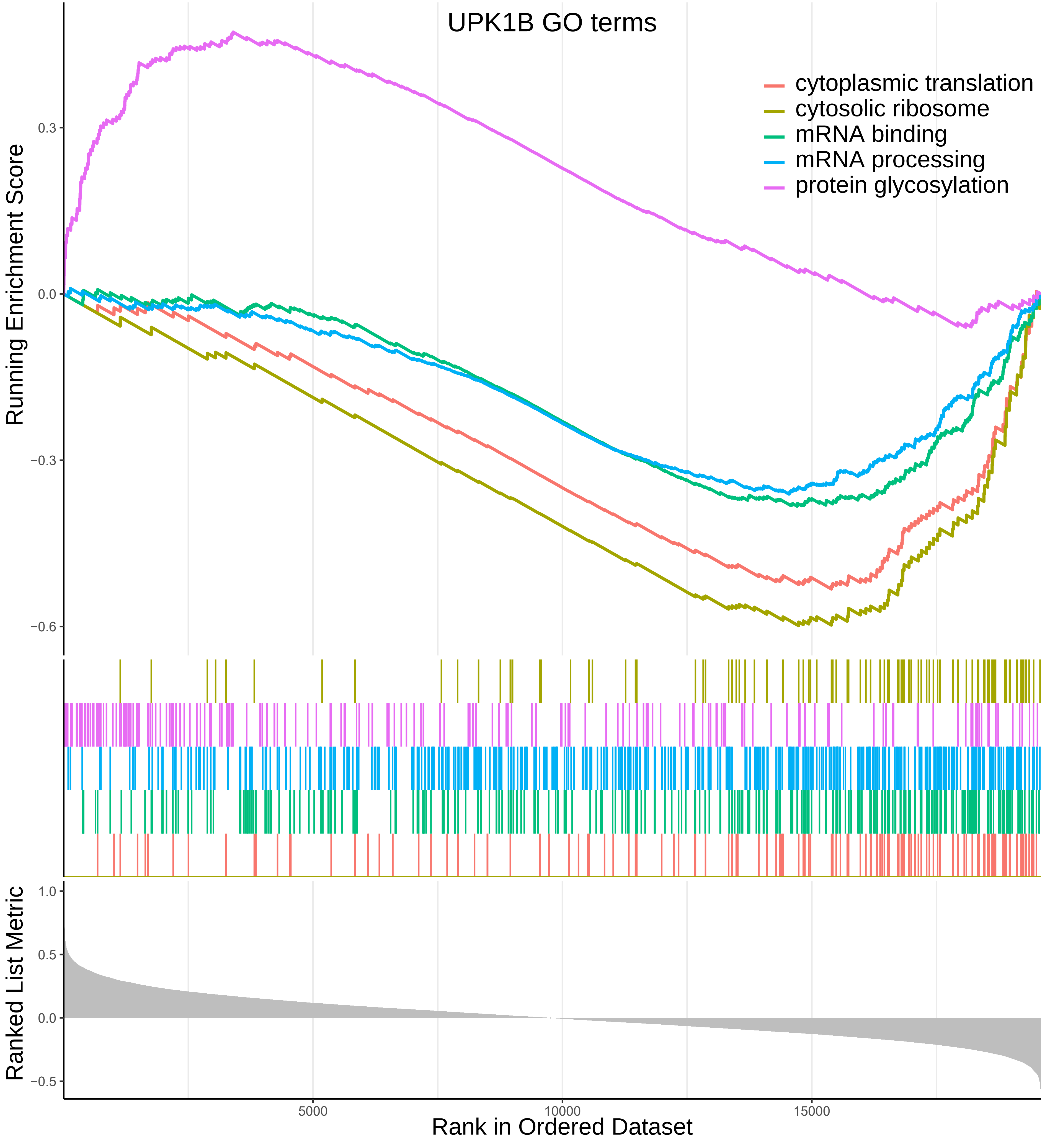
UPK1B GO terms
cytoplasmic translation
cytosolic ribosome
mRNA binding
mRNA processing
protein glycosylation
Running Enrichment Score
0.3
0.0
−0.3
−0.6
Ranked List Metric
1.0
0.5
0.0
−0.5
5000
10000
15000
Rank in Ordered Dataset

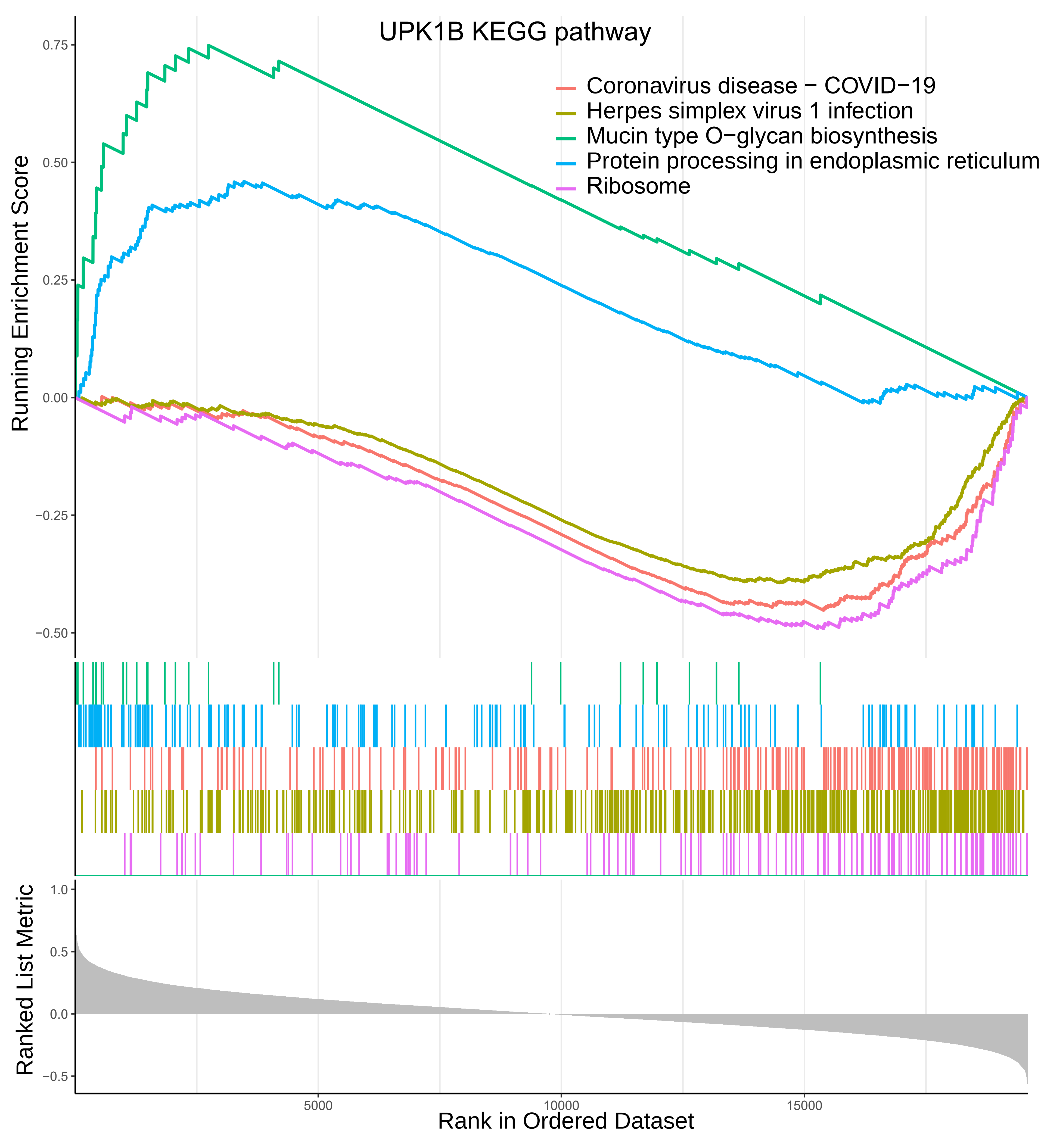

UPK1B KEGG pathway
Coronavirus disease − COVID−19
Herpes simplex virus 1 infection
Mucin type O−glycan biosynthesis
Protein processing in endoplasmic reticulum
Ribosome
Running Enrichment Score
0.75
0.50
0.25
0.00
−0.25
−0.50
Ranked List Metric
1.0
0.5
0.0
−0.5
5000
10000
15000
Rank in Ordered Dataset

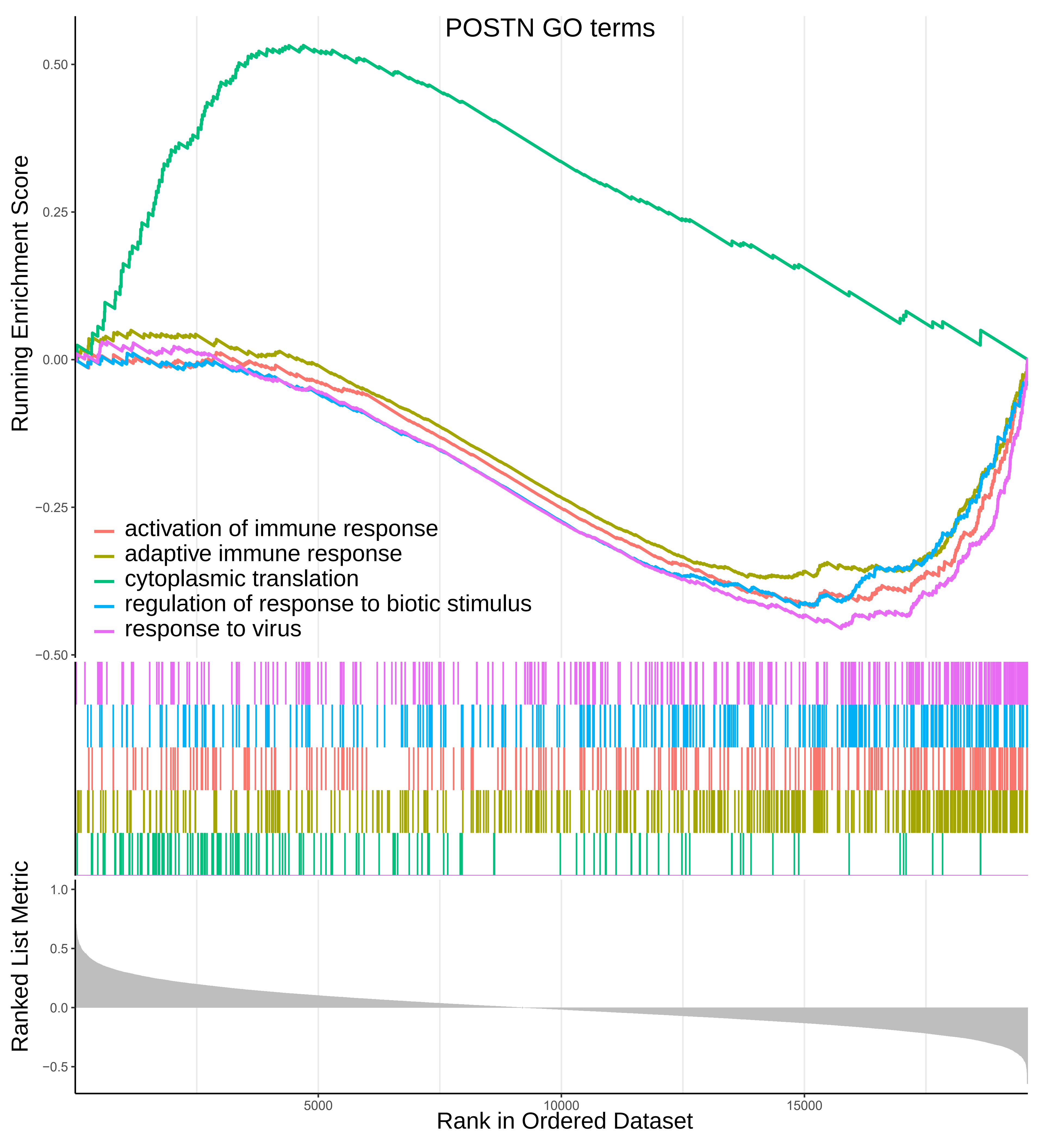
POSTN GO terms
Running Enrichment Score
0.50
0.25
0.00
−0.25
−0.50
activation of immune response
adaptive immune response
cytoplasmic translation
regulation of response to biotic stimulus
response to virus
Ranked List Metric
1.0
0.5
0.0
−0.5
5000
10000
15000
Rank in Ordered Dataset

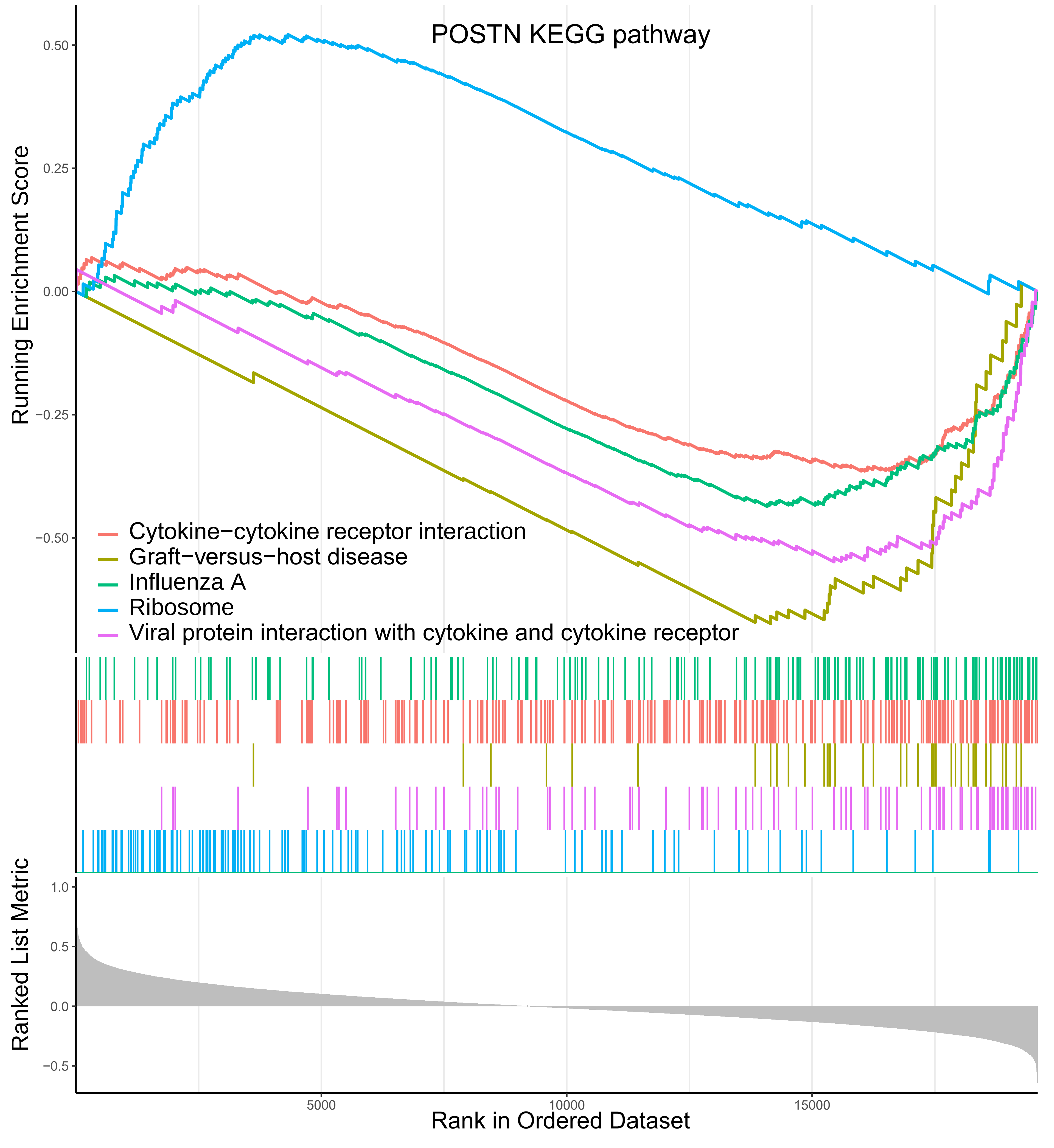

POSTN KEGG pathway
Running Enrichment Score
0.50
0.25
0.00
−0.25
−0.50
Cytokine−cytokine receptor interaction
Graft−versus−host disease
Influenza A
Ribosome
Viral protein interaction with cytokine and cytokine receptor
Ranked List Metric
1.0
0.5
0.0
−0.5
5000
10000
15000
Rank in Ordered Dataset

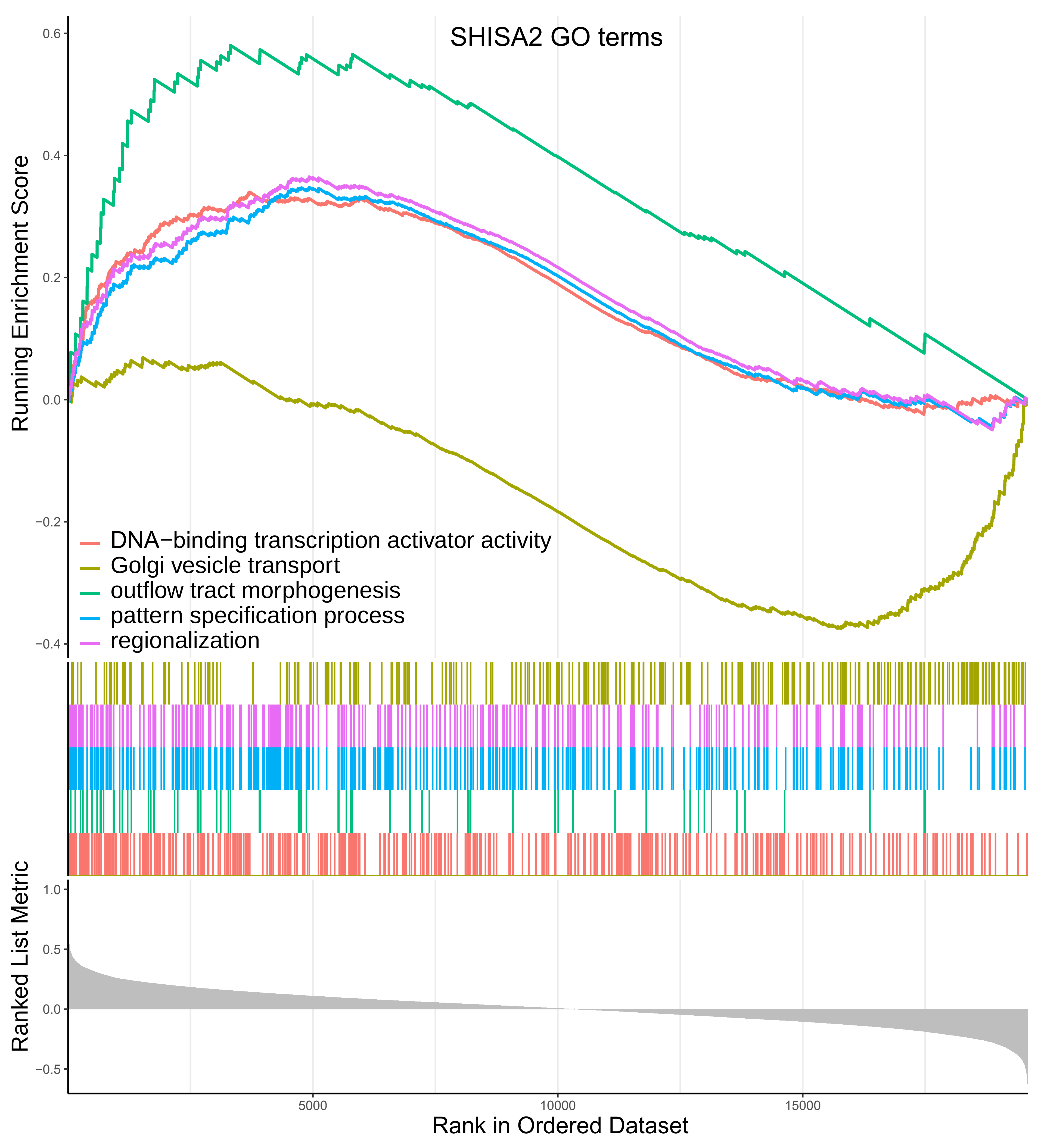

SHISA2 GO terms
Running Enrichment Score
0.6
0.4
0.2
0.0
−0.2
−0.4
DNA−binding transcription activator activity
Golgi vesicle transport
outflow tract morphogenesis
pattern specification process
regionalization
Ranked List Metric
1.0
0.5
0.0
−0.5
5000
10000
15000
Rank in Ordered Dataset

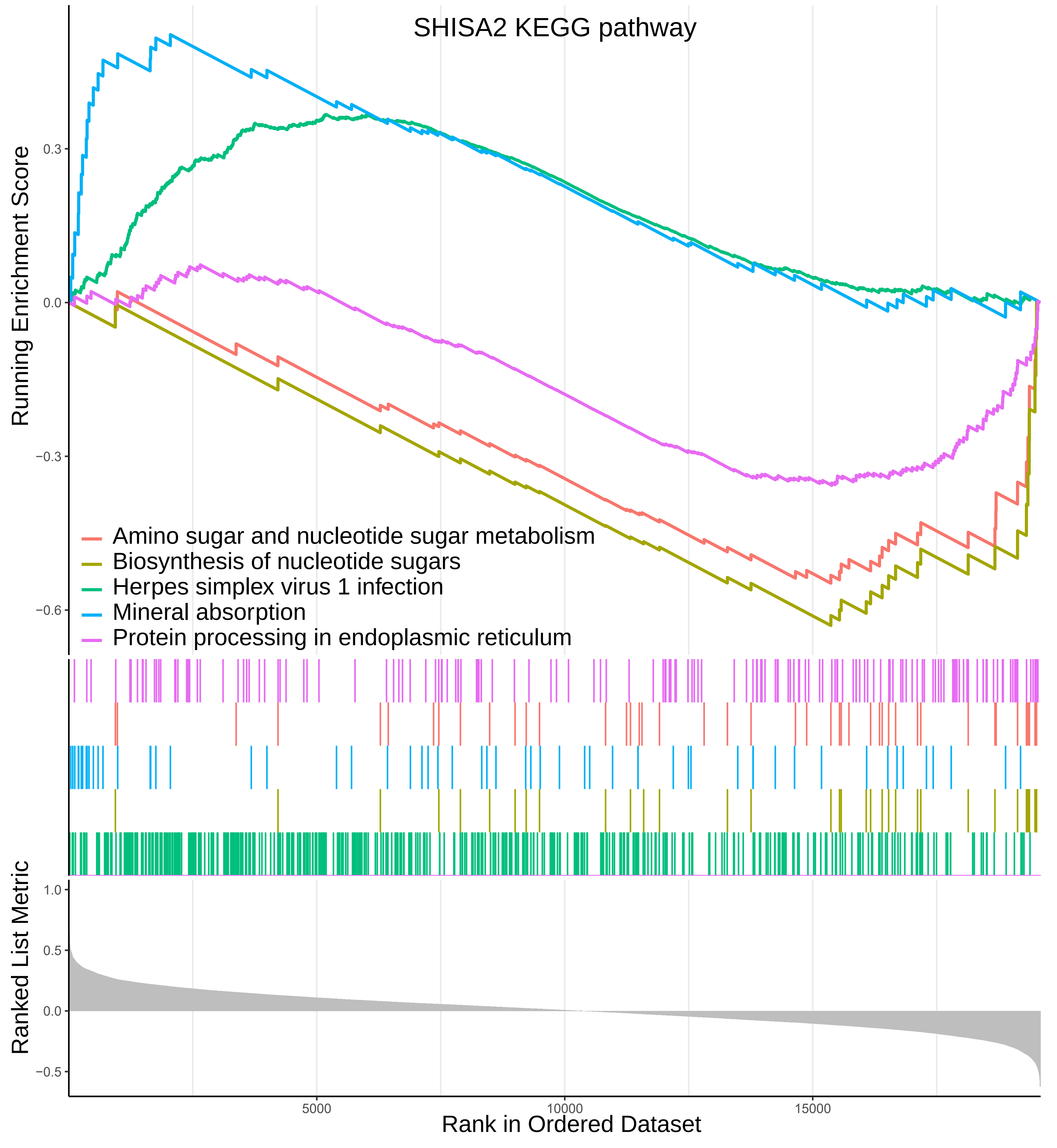
SHISA2 KEGG pathway
Running Enrichment Score
0.3
0.0
−0.3
−0.6
Amino sugar and nucleotide sugar metabolism
Biosynthesis of nucleotide sugars
Herpes simplex virus 1 infection
Mineral absorption
Protein processing in endoplasmic reticulum
Ranked List Metric
1.0
0.5
0.0
−0.5
5000
10000
15000
Rank in Ordered Dataset
